# Multi-mode hybrid micro-gripper for micro-manipulation and micro-assembly

**DOI:** 10.1016/j.ohx.2026.e00850

**Published:** 2026-10-14

**Authors:** Gianmauro Fontana, Nicola Iacono, Gabriele Papadia, Simone Pio Negri

**Affiliations:** a Institute of Intelligent Industrial Technologies and Systems for Advanced Manufacturing, CNR-STIIMA, Via A. Corti 12, Milan, 20133, Italy; b Department of Mechanical and Industrial Engineering, University of Brescia, Via Branze 38, Brescia, 25123, Italy; c Department of Innovation Engineering, University of Salento, Via per Monteroni, Lecce, 73100, Italy; d Institute of Intelligent Industrial Technologies and Systems for Advanced Manufacturing, CNR-STIIMA, Via P. Lembo 38/F, Bari, 70124, Italy

**Keywords:** Hybrid micro-gripper, Vacuum micro-gripper, Two-finger micro-gripper, SMT assembly, Closed-loop control of stepper motor, PID controller

## Abstract

This work presents the design, implementation, and validation of a multi-mode hybrid micro-gripper for robotic micro-manipulation and assembly in smart manufacturing applications. The device combines vacuum-and friction-based grasping technologies in a compact, lightweight design, enabling accurate and reliable handling of micro- and meso-scale Surface Mount Technology (SMT) components. The system supports three operational modes: friction, vacuum, and a hybrid grasping that simultaneously engages both technologies. The gripper features a modular architecture and can be manufactured with additive-manufactured technology and low-cost commercial components. Each finger of the friction grasping is actuated by a miniature linear stage equipped with a stepper motor, controlled through a closed-loop motion algorithm based on a PID regulator and a linear potentiometer for position feedback. A two-state control manages the vacuum supply to the suction cup and the pneumatic cylinder, which enables hybrid grasping. All three operational modes were experimentally validated, demonstrating high accuracy and reliability in manipulating different SMT components with dimensions in the range of 0.5 × 0.5 mm to 8 × 8 mm. Extensive experimental tests were performed for friction grasping to assess positioning accuracy, motion synchronisation, disturbance rejection, and stall detection, achieving positioning errors below ± 50 µm and synchronisation errors under 30 ms.

## Graphical abstract

**Figure:**
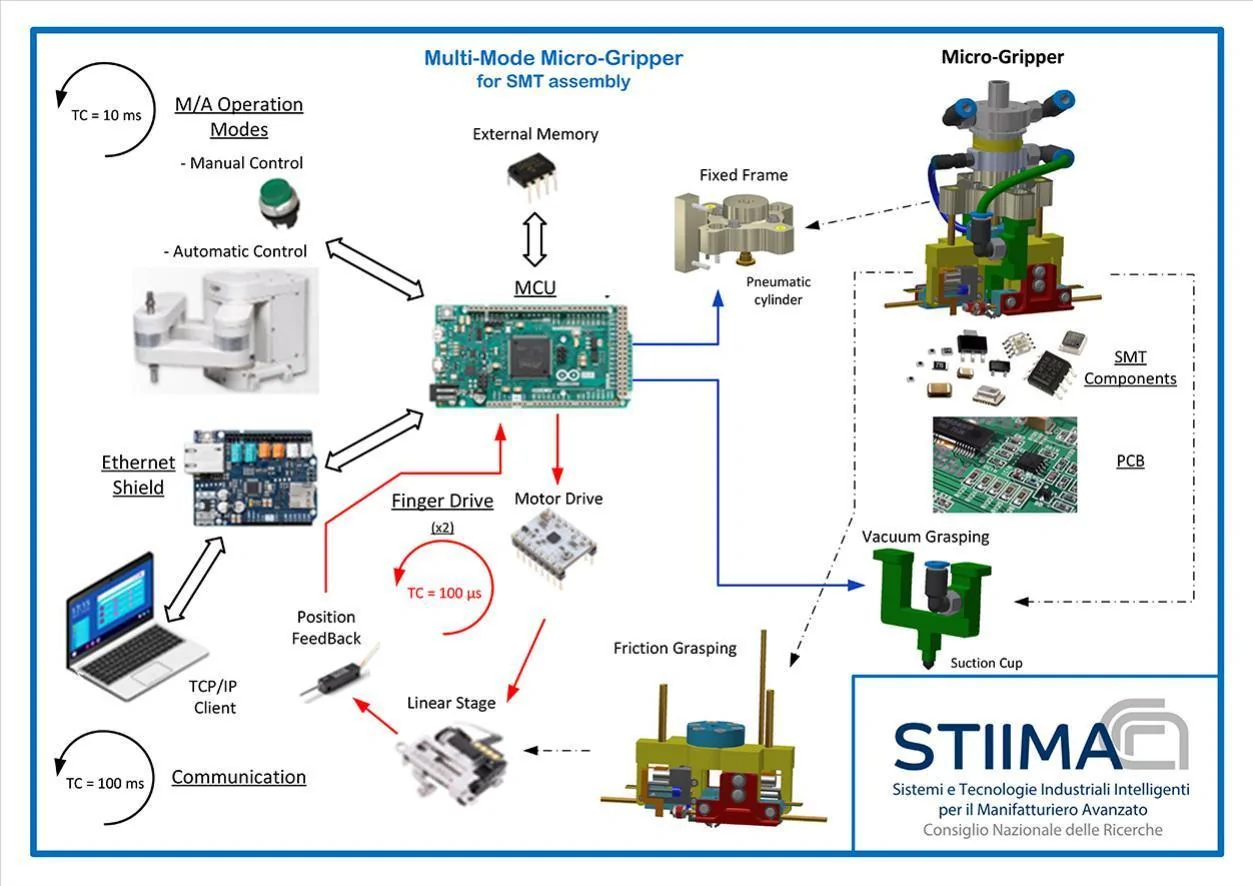

**Specifications table:** 

Hardware name	• Multi-mode hybrid micro-gripper for micro-manipulation and micro-assembly
Subject area	• Engineering and materials science
Hardware type	• Mechanical engineering and materials science
Open source license	Creative Commons Attribution 4.0 International. To view a copy of this license, visit https://creativecommons.org/licenses/by/4.0.
Cost of hardware	562.66 € (+ 750 € for automatic tool changer)
Source file repository	https://doi.org/10.17632/bpsmdb9nkf.1

## 1 Hardware in context

The current trend of miniaturisation in the electronics field, driven by the need to integrate more functionality within smaller devices, poses considerable challenges for automated assembly processes. Moreover, the increasing demand for product customisation and small-batch production requires flexible and adaptable manufacturing systems [1].

The robotic assembly of printed circuit boards (PCBs) requires accurate and reliable manipulation of a wide variety of Surface Mount Technology (SMT) components. These components can range from micro-scale, e.g., MEMS or passive micro-components, to meso-scale, e.g., integrated circuits or connectors, and they vary in geometry and materials. The assembly of SMT components necessitates precise placement on the PCB pads, followed by soldering. Typically, the required placement and soldering accuracy for 0201 (metric scale) SMT passive components range from 100 to 50 micrometers, depending on the adopted class of the IPC-A-610D standard [2] that defines the criteria for the electronic assembly.

Automated PCB assembly presents significant challenges, demanding high precision, reliability, and throughput while also ensuring flexibility and cost-effectiveness [3]. Therefore, high-throughput, flexible and accurate automated assembly work-cells equipped with robots, grasping tools, soldering devices, sensing systems and advanced control strategies are essential to cope with the stringent new requirements of PCB assembly [4].

The growing need to handle a wide range of different SMT components, often extremely small, is leading to the development of new, accurate and flexible technologies for SMT manipulation.

Manufacturers and researchers are actively exploring and developing new reconfigurable manipulation methods and grasping tools (i.e. grippers to be mounted on the robot end-effector) to enhance the performance of automated work-cells.

Indeed, conventional grasping tools are limited by micro-scale adhesion forces (e.g. electrostatic interactions, van der Waals forces, and capillary effects) due to micro-component dimensions to be manipulated [5] and by the heterogeneity of SMT components, which can vary in dimensions, shapes, and materials [6]. In electronics manufacturing, robotic assembly systems often adopt many grippers, each dedicated to specific micro- or meso-components. Adopting a wide set of tools, that are also capable of micrometric resolution, introduces several issues, resulting in a more complex, expensive, and time-consuming process and setup. Indeed, this approach requires frequent tool changes. These tool changes lead to extended setup and task times, reducing production efficiency and increasing costs, especially for small-batch PCB manufacturing with a high rate of different components. Therefore, adopting grippers designed for specific component types limits the throughput and flexibility of the robotised work-cell [7]. The most commonly used grippers in this sector are vacuum grippers, such as rubber tip grippers by Micro-Mechanics [8] and friction grippers with two fingers, such as the MPG series grippers by Schunk [9]. Both types are usually designed to manipulate a specific component or a limited range of component types. Over the past few decades, growing interest from the scientific and industrial communities in grasping technologies has led to the design and development of new methods and tools for PCB assembly with micro- and meso-scale SMT components. These advancements aim to address current limitations and include the development of various gripper types, such as vacuum, friction, electrostatic, and capillary grippers [10].

Vacuum grasping exploits the suction force (i.e., the pressure difference between the gripper and the atmosphere) to grasp objects. It is particularly effective for handling flat or slightly curved components with smooth surfaces. These grippers perform accurate and reliable assembly with minimal positioning errors, ensuring stable and safe grasping and precise releasing [11]. They consist of one or more nozzles/cups connected to one or more vacuum ejectors/pumps. [12]. These grippers can implement an open or closed-loop pressure control strategy. The bandwidth of these systems is very high, so the gripper response time is very low. Vacuum grippers are designed to manipulate SMT components with specific dimensions and properties according to the dimensions, shape and material of the nozzle and the supply negative pressure [5], [13]. They manipulate the parts from above through a monolateral vertical force, and then they can manufacture PCBs with a high density of components. Vacuum grippers can be easily and cost-effectively miniaturised, allowing to handle micro-components as small as 100 μm with an accuracy of less than 10 μm [14]. Specific solutions are needed at the micro-scale to support the releasing phase due to the effects of adhesion forces between the micro-object and the vacuum micro-gripper. Different releasing strategies (e.g. vibration [15] or positive pressure [16]), releasing integrated mechanisms (e.g. passive system [5]), or absorption models to calculate the adhesion forces and control the releasing operations [14] can be found in academic literature. However, by downsizing the gripper dimensions, it can be easily occluded, losing reliability.

Friction grippers consist of two or more fingers that move to grasp and release SMT parts. They can be actuated using various methods, including pneumatic [17], electric [18], [19], [20], electromagnetic [21], shape memory alloys (SMAs) [22], [23], or piezoelectric actuators [24], [25]. Response times of the gripper depend on the bandwidth of the adopted actuation, driving and control systems. They can be very high for pneumatic and electric actuators and very low for SMA. The vacuum micro-grippers can be designed to manipulate a wide range of components with different dimensions and shapes. Typically, the actuators of the gripper's fingers are controlled by adopting open-loop positioning strategies, in particular for the pneumatic actuators and stepper electric motors. A more accurate positioning of the fingers can be achieved by implementing closed-loop position control strategies. Usually, these grippers are not suitable for very fragile parts unless a closed-loop force control is implemented for finger movements. Additionally, they have exhibited undesired sticking effects, which can prevent the release or accurate placement of micro-parts with dimensions under 800 µm without adopting suitable releasing strategies [26].

Electrostatic grippers exploit the attraction between charged plates to pick parts. These grippers are compact and lightweight, allowing the manipulation of components with dimensions up to 10 μm [27]. The bandwidth of these systems is high, but they require high operating voltages, commonly ranging from several tens to several hundreds of volts, depending on the electrode configuration and the required grasping force. Moreover, they can be influenced by external electric fields [28], [29], [30]. For these reasons, electrostatic grippers are not widely used for PCB assembly.

Capillary grippers utilise a small liquid droplet on the gripper surface to create a liquid bridge with the part to manipulate. This bridge, along with the meniscus (i.e. the curvature of the bridge on the liquid surface), generates a capillary force. This force is strong enough to grasp and hold micro-components with dimensions up to 10 μm [31]. To release the component, the gripper simply moves away, breaking the liquid bridge and placing the component accurately in the desired position [32], [33], [34]. The surfaces of the gripper and the liquid used to create the bridge should be designed according to the specific SMT component to manipulate. Due to the minimal mechanical stress applied, these grippers are suitable for fragile components. These grippers are moved at a slow speed to achieve reliable and accurate grasping, holding and releasing. The gripper response times are high. Moreover, their performance can be affected by the presence of contaminants or varying environmental conditions [35]. Therefore, this grasping technique is not widely used in PCB assembly since applying or creating a capillary bridge on SMT micro-components is not always possible.

Hybrid micro-grippers have been developed, combining different grasping strategies or actuation methods to enhance manipulation capabilities at the micro/meso scale. In [36], an electrostatically actuated micro-gripper with two fingers featuring a post-assembly self-locking mechanism that provides external force without continuously applying the driving signal was described, illustrating how mechanical features can be combined with micro-actuators to increase holding robustness for manipulating objects of sizes from 0 to 100 μm. A hybrid electrothermal micro-gripper designed to effectively pick and hold micro parts with sizes in the range from 110 to 160 μm was presented in [37], incorporating V-shaped and U-shaped electrothermal micro-actuators. In [38], a comb-drive electrostatic micro-tweezer equipped with an integrated vacuum tool was proposed to manipulate micro-objects (i.e., dimensions ranging from 100 to 200 µm). In [39], a multi-mode hybrid micro-gripper was developed for assembling SMT components ranging in size from 0.5 to 8 mm, exploiting both vacuum and two-finger friction grasping.

Integrating sensors, such as force sensors [40], [41], vision systems [13], [42], [43], and position/speed sensors into the mechanical structure of the designed micro-grippers or the robotic system where they are used allows the implementation of closed-loop control, as well as supervision and inspection algorithms, enhancing the manipulation accuracy, flexibility and autonomy. These sensors can provide feedback on the gripper state (e.g. the pose of the fingers for friction grippers or the nozzle pressure for vacuum grippers), grasping force, contact detection, object pose, and object identification, enabling more accurate manipulation, especially for delicate and fragile micro-parts. External vision systems in PCB assembly robotised work-cells typically provide the component's pose, which the robot uses to compensate orientation and position errors, ensuring accurate placement.

Due to the high accuracy demand, the calibration of sensing systems should ensure high measurement performance with high resolution [44], [45], allowing the exchange of information with the robot in order to achieve the most accurate component positioning (i.e., position and orientation) and to apply the appropriate assembly force.

This article presents the design, implementation, and experimental validation of the novel hybrid microgripper based on our previous work [39]. The proposed tool is specifically designed to manipulate a wide range of micro- and meso-scale components with different dimensions, shapes, and properties. Indeed, it is conceived to handle the most used SMT parts typically assembled on PCBs, eliminating the need for frequent tool changes. It can operate in three selectable operational modes, vacuum, friction, and hybrid grasping, to adapt to diverse assembly tasks, including collaborative robotics operations such as operator-assisted soldering. Closed-loop motion control of the miniature linear stages ensures high finger positioning accuracy, and the gripper is equipped with interchangeable micro-suction cups to cover its entire dimensional range of application. Its lightweight, low-cost, and modular design allows for seamless integration into robotic work-cells. The tool aims to enhance the flexibility, efficiency, and positioning accuracy of robotic PCB assembly processes.

## 2 Hardware description

The multi-mode micro-gripper, shown in Fig. 1, exploits two technologies to achieve high flexibility in SMT component assembly. These technologies are vacuum and friction grasping. The vacuum technique uses a micro suction cup to pick the component from the top, i.e. monolateral vertical grasping. The friction grasping is achieved through two parallel mini-fingers actuated to operate against each other along a horizontal axis to grip the component from the side (i.e., bilateral horizontal grasping).

**Fig. 1:**
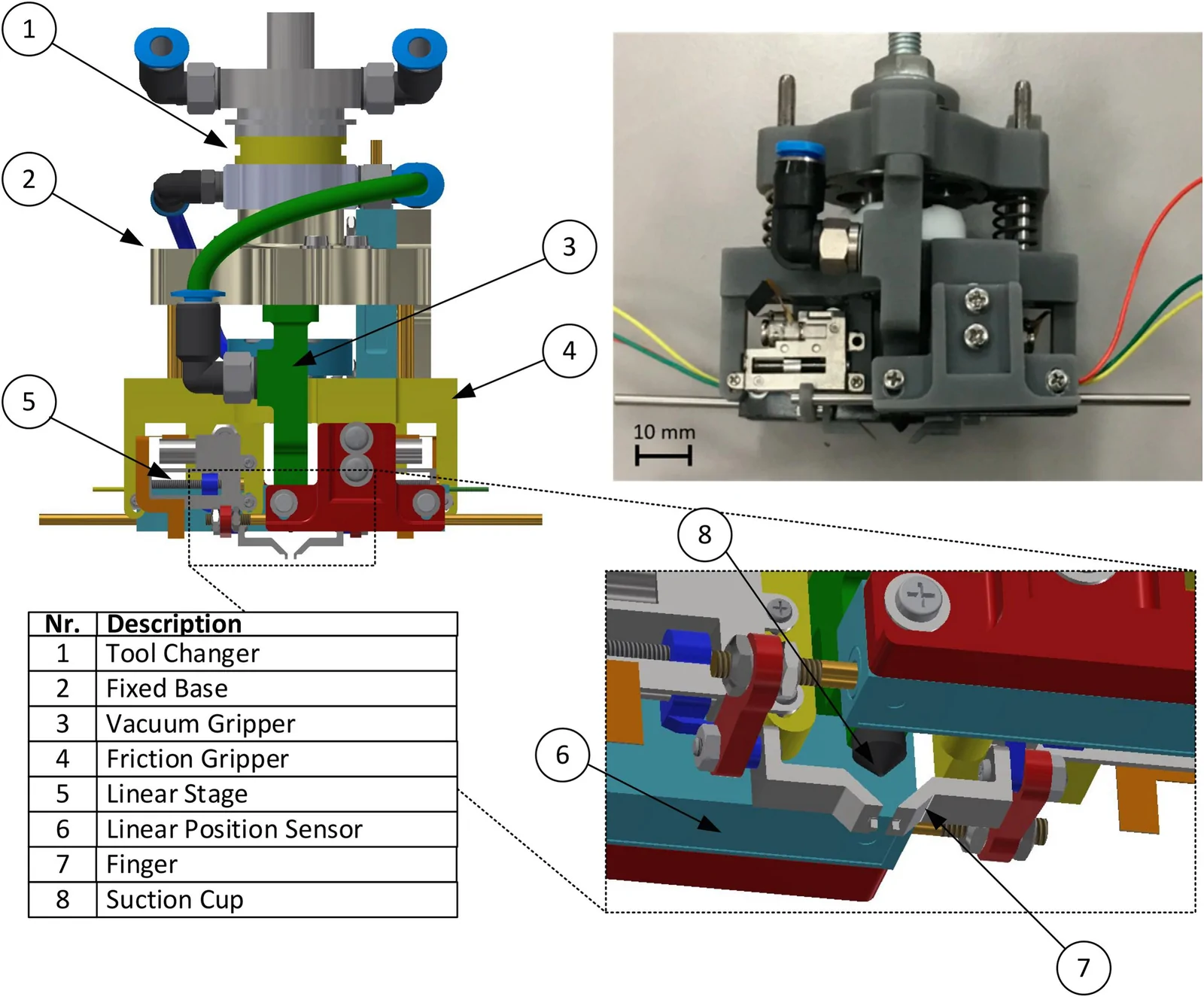
CAD design of the multi-mode micro-gripper with the detail of the grasping technologies and front view of the developed prototype.

Due to its multi-mode operations, it was designed to manipulate electronic components with a planar dimension in the range from 0.5 x 0.5 mm to 8 x 8 mm and with a variable height from 0.3 mm to 5 mm.

Moreover, the micro-gripper was conceived to allow human-robot or robot-robot cooperation during the soldering process. Indeed, exploiting the hybrid operational mode (i.e., where both technologies are used simultaneously), the micro-gripper can firmly hold the components in the soldering place while an operator or an automatic system performs the soldering task. Indeed, the gripper was designed to overcome the forces exerted by the soldering tool in the SMT assembly task. These forces can act along both vertical and horizontal axes, ranging from 0.05 to 0.2 N. The gripper fingers were produced by additive manufacturing, specifically utilising the high-temperature resin by Formlabs. This material can withstand soldering temperatures up to 240° (refer to Section 4).

The micro-gripper is composed of three main sub-assemblies and the automatic tool changer. The tool changer is a standard commercial component, and it does not belong to the micro-gripper in the strict sense. The sub-assemblies are listed below, along with the corresponding names of CAD files (refer to Section 3) in brackets:

1. the fixed base with the single-acting pneumatic cylinder (i.e. Base9);
2. the vacuum grasping tool, i.e. the suction cup (i.e. CentralPart12);
3. the friction grasping tool, i.e. the two fingers (i.e. MobileFrame15).

The fixed base supports the vacuum grasping tool, which is fixed, and the friction grasping tool, which can move in the vertical direction driven by the pneumatic cylinder (i.e. the vertical axis).

The vacuum grasping sub-assembly includes a holder for mounting interchangeable miniature rubber suction cups with different internal and external diameters ranging from 0.1 to 3 mm, allowing the manipulation of components of various sizes. The gripper prototype was equipped with a rubber suction cup with an inner diameter of 0.4 mm and an external diameter of 0.6 mm (i.e. R7M-0050-1 by Micro-Mechanics).

To allow flexibility in component manipulation, the fingers of the friction grasping tool were designed to be interchangeable. Two versions were designed: the former presents an L shape, and the latter presents a T shape, with both having a grasping surface of 0.4 × 0.4 mm. For the gripper prototype, the L-shaped fingers were manufactured. The two fingers can move in the horizontal direction through two low-cost miniature linear stages (i.e. horizontal axes) with a stroke of 9.1 mm and a linear full step of 25 µm. Each linear stage is actuated by a 2-phase stepper motor and equipped with a transmission system for reducing and transforming the motion from rotary to linear by gears coupled with a lead screw and nut. Since the micro-gripper is designed to operate as an end-effector of a robot, it was equipped with an automatic tool changer (i.e. quick-release interface), attached to the fixed base, allowing to connect and disconnect the gripper to the robot automatically. The automatic tool changer is not mandatory, and it can be replaced by a fixed interface.

The main advantage of the micro-gripper is the possibility of operating either by friction grasping, vacuum grasping, or both. Moreover, these functionalities are realised by a lightweight and compact design.

Compared to other commercial grippers, the micro-gripper has the following advantages:

• it can handle a wide range of SMT components with different dimensions, shapes, and properties;
• it was also designed to perform collaborative soldering tasks;
• its commercial components are low-cost;
• most of its parts are realised by additive manufacturing, and only a few components need to be manufactured using cutting technology.

### 2.1 Mechanical Design

This section illustrates the mechanical design for the horizontal (i.e. linear stages) and the vertical (i.e. pneumatic cylinder) axes of the fingers. Moreover, the grasping forces that the gripper can exert in its three operational modes are analysed.

Fig. 2a shows the forces and the moments acting on the electronic component during the friction grasping (*F_T_* and *F_N_*, in green) and their reactions on the horizontal axes (F_V_, F_H_ and M). Only one axis is shown, and the other is dual (i.e., *F_N_* and *F_H_* have opposite directions).

**Fig. 2:**
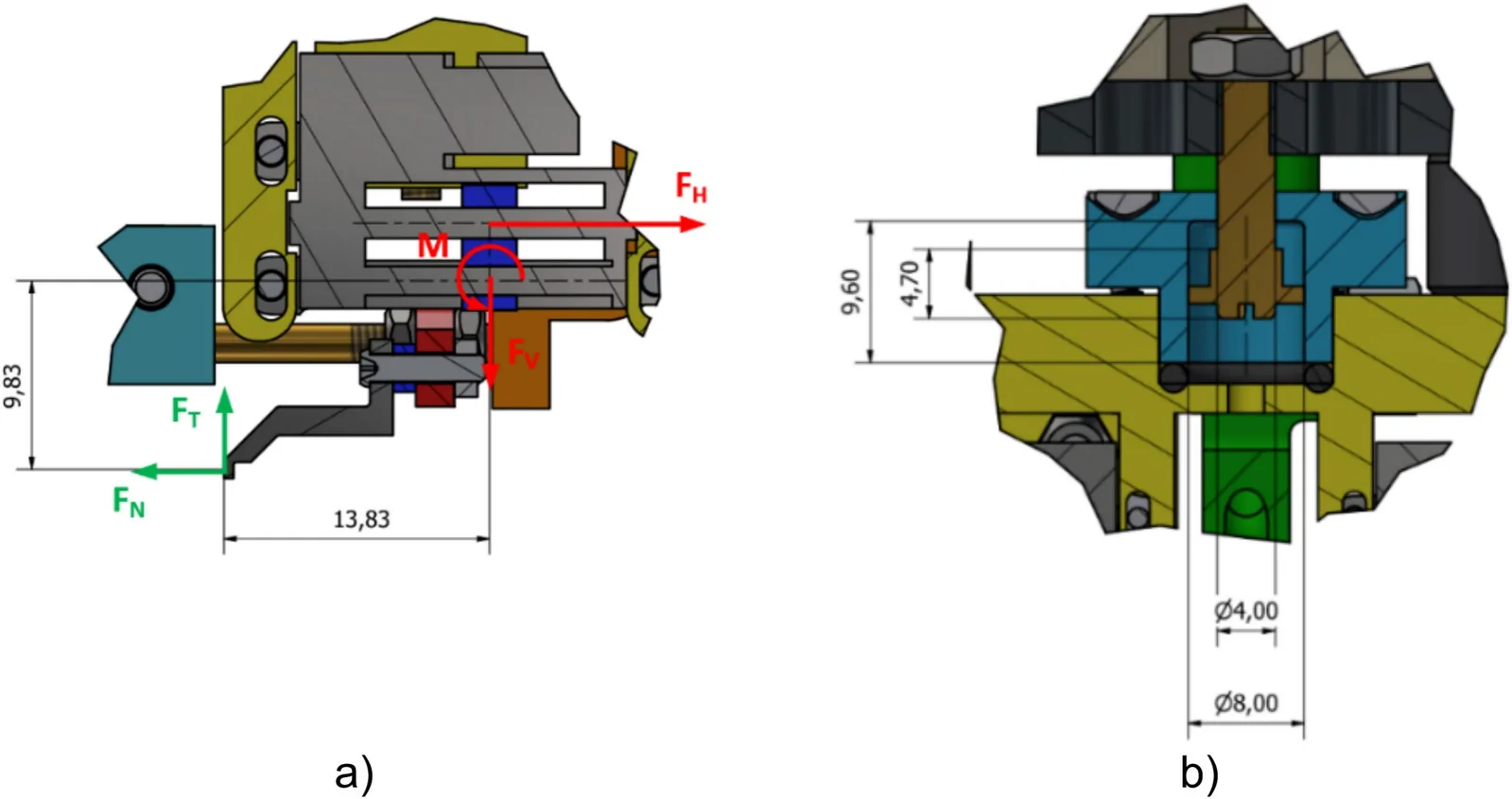
Design of (a) horizontal axes for friction grasping and (b) the vertical axis.

It is possible to find the reaction forces on the finger axes, starting from the forces applied to the component. The force system acting on the finger axes can be defined as:

(1) $$\left\{\begin{array}{c} F_{H}=F_{N}\\ F_{V}=F_{T}\\ M=F_{N}∙9.83+F_{T}∙13.83 \end{array}\right)$$

Where:

• *F_N_* and *F_T_* are the normal and transversal forces acting on the component to grasp. An evaluation of them is in the range of 0.05 – 0.2 N, which represents the forces exerted by the soldering tool during the soldering process (in the case of transversal force, the component weight force is not considered because it is negligible);
• *F_H_* and *F_V_* are the horizontal and vertical forces acting on the linear axis. *F_H_* is supported by the motor through the screw of the stage, while *F_V_* is supported by the bottom shaft of the stage through its sleeve bearings;
• *M* is the moment applied to the stage bottom shaft by *F_N_* and *F_T_*. For *F_N_* = *F_T_* = 0.2 N, *M* = 4.732 Nmm;
• 9.83 mm and 13.83 mm are the force arms' lengths for *F_N_* and *F_T_*_,_ respectively.

Each of the two linear axes chosen for the two fingers ensures an *F_H_* of 0.2 N and an *F_V_* of 0.2 N. They have a stroke of 9.1 mm and a linear step of 25 µm when controlled in full step.

Fig. 2b shows the section view of the pneumatic cylinder. It is worth to note that it is not equipped with piston rod seals to reduce friction and the stick-slip effect. The vertical axis design was performed, considering a maximum air pressure for the cylinder of 6 bar.

The useful cylinder section for calculating the lifting force is:

(2) $$A=\pi \left(R^{2}-r^{2}\right)=\pi \left(4^{2}-2^{2}\right)=37.699{mm}^{2}\approx 0.37{cm}^{2}$$

The force exerted at 6 bar is:

(3) $$F=p∙A=6bar∙0.37{cm}^{2}=22.2N$$

The calculated lifting force is high enough to lift the gripper mobile part (i.e. the friction grasping tool), overcoming its weight force and the elastic force exerted by the return springs of the single-acting pneumatic cylinder. These springs ensure the return to the bottom position of the gripper mobile part when the atmospheric pressure is restored inside the cylinder. Otherwise, the return relies only on the weight force of the mobile part.

Manufacturing tolerances are another crucial aspect of the vertical axis design. There are two opposed specifications: achieving accurate positioning while minimising friction as much as possible. This issue affects the tolerance requirement between the piston rod (i.e. brown part in Fig. 2b) and the cylinder (i.e. blue part in Fig. 2b). A little backlash between these two elements was introduced to achieve the best trade-off between accurate positioning and low friction. For this reason, a mobile coupling with an ISO dimensional tolerance of H7/h6 was designed. Moreover, a little air leaks from the gap, ensuring an air bearing between the cylinder and the piston rod.

The micro-gripper can operate in three modes: friction, vacuum, and hybrid grasping.

The maximum dimensions of an SMT component that the micro-gripper can grasp are 8 × 8 × 3 mm, corresponding to a volume of 192 mm^3^. Supposing a material density (i.e. *ρ*) of 3.74 g/cm^3^, the component weight force (i.e. *W*) is 7.3 mN.

To design the friction-based grasping mechanism, a specific static friction coefficient μ must be assigned. Determining a precise coefficient is inherently complex and uncertain, as friction depends on several factors, including surface, environmental conditions, and the specific type of electronic component being grasped. A rigorous estimation would require an exhaustive tribological investigation, which is beyond the scope of this work. Therefore, a first-order approximation has been adopted, assuming a friction coefficient of μ = 0.5 for the polymer–metal contact, a representative rounded-up average value within the typical range reported for such material pairs (i.e., 0.1–0.8). Considering a closing force (i.e. *F_c_*) of 0.2 N for each finger, the maximum weight force of the component that the gripper can grasp and hold with only friction grasping is:

(4) $$F_{f}=2∙F_{c}∙\mu =0.2N$$

Regarding the vacuum grasping, with a negative pressure (i.e *p*) of −0.75 bar and a suction cup diameter (i.e *d*) of 0.4 mm, resulting in an effective grasping area (i.e. *A*) of 0.126 mm^2^, the corresponding suction cup force is:

(5) $$F_{v}=p∙A=-0.01N$$

The term *F_v_* contributes marginally to the total force, but it is relevant for the analytical design model. Indeed, for example, the vacuum cup diameter can change from the actual 0.4 mm to 1 mm, and in this latter case, the vacuum force increases from the actual -0.01 N to -0.59 N, which is of the same order of magnitude as the friction grasping force.

Adding the two contributions, the total force that the gripper can exert in hybrid mode is:

(6) $${F_{t}=F_{f}+|F}_{v}|=0.21N$$

Fig. 3 shows an overview of the micro-gripper and its main sub-assemblies, highlighting the maximum forces that can be reached during friction and vacuum grasping.

**Fig. 3:**
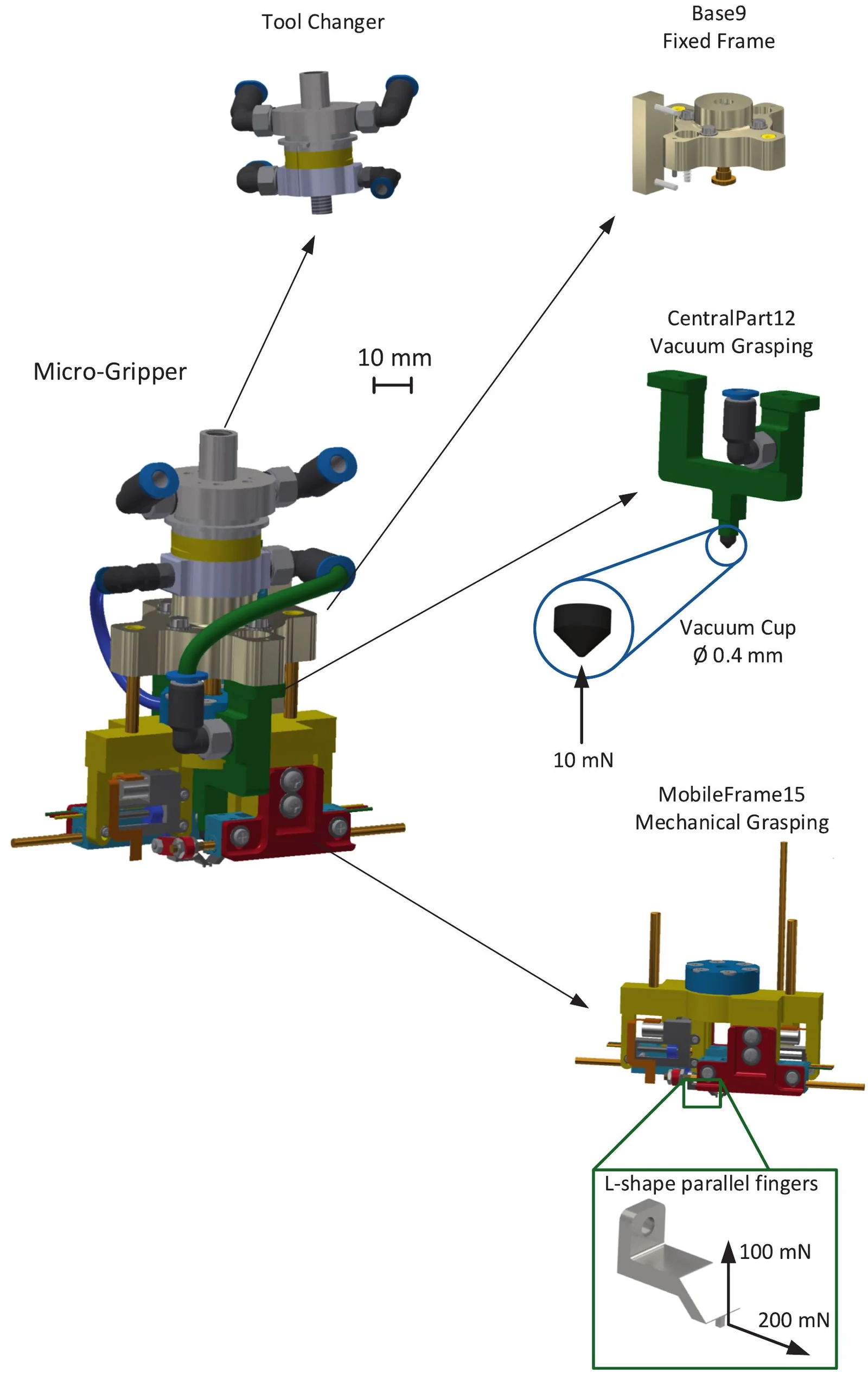
Overview of the micro-gripper and its main sub-assemblies. The maximum grasping forces are shown for the vacuum and friction operational modes.

### 2.2 Control System

The functional block scheme of the gripper control system is shown in Fig. 4 (refer to Section 3 for the electronic schematics).

**Fig. 4:**
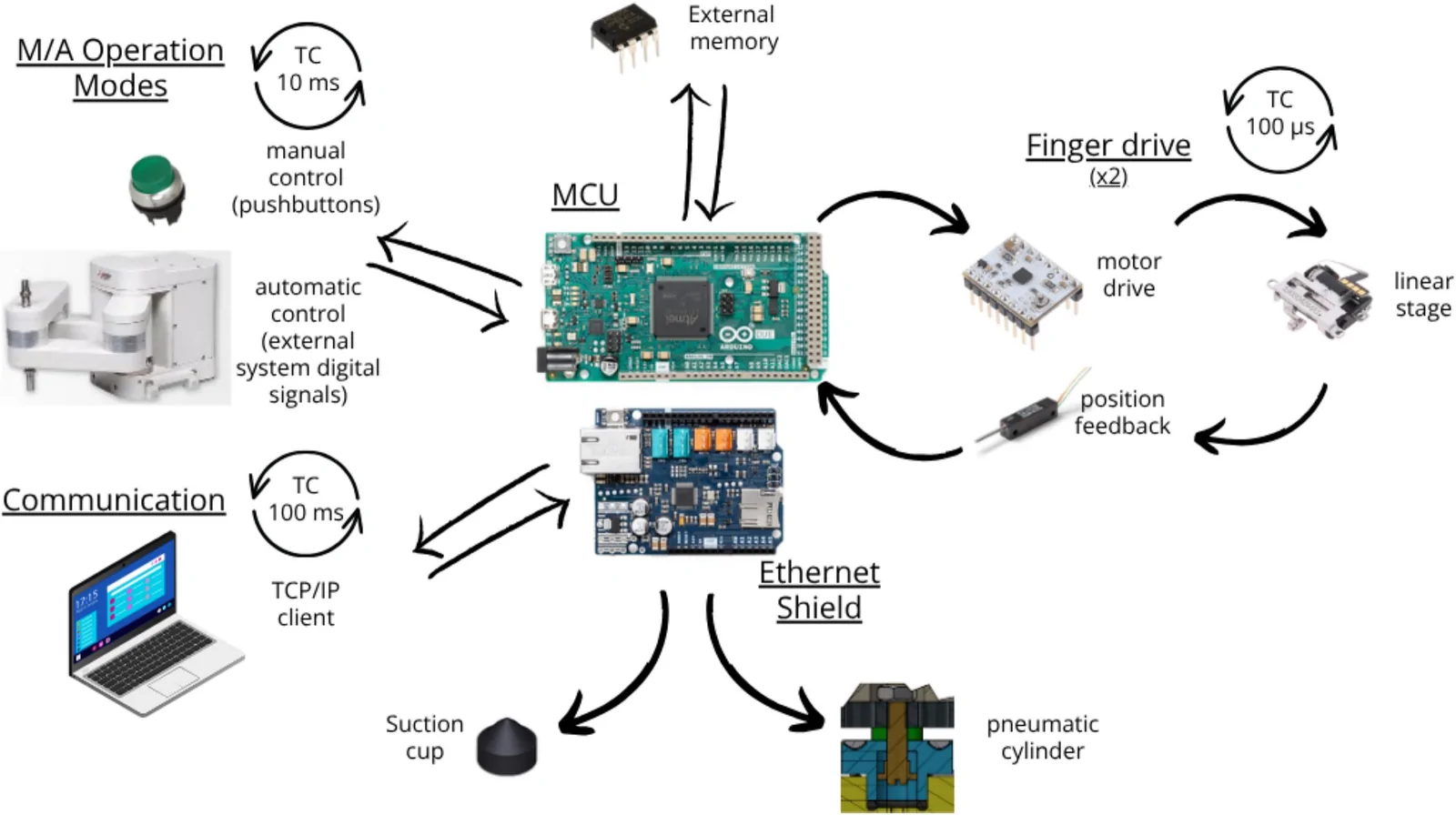
Functional scheme of the micro-gripper controller.

The controller was designed to manage all the gripper functionalities. The Arduino Due microcontroller (i.e. MCU) is the core of the control system, executing the control firmware stored in its embedded flash memory. It is based on the Atmel SAM3X8E ARM Cortex-M3 processor, with a clock speed of 84 MHz, a 12-bit ADC with up to 16 input channels, two 12-bit DAC outputs, a flash memory (i.e. non-volatile memory) of 512 KB for program storage, and a SRAM (i.e. volatile memory) of 96 KB for runtime data.

Regarding the friction grasping technology, the MCU enables the implementation of both open- and closed-loop motion control strategies for positioning the two linear stages and their corresponding fingers. Each finger motion system is composed of three main elements. The micro-linear stage is directly connected to the finger, allowing for its precise positioning. The STSPIN220 Low-Voltage Stepper Motor Driver (by Pololu) is used to drive the stepper motor of the linear stage with a STEP/DIR logic. An MM10 linear potentiometer (by Megatron) is used as position feedback of the gripper finger.

Two digital outputs of the microcontroller are used to perform two-state (i.e., on/off) control of the suction cup and the pneumatic cylinder, enabling the vacuum activation and the upward motion of the fingers, respectively. These outputs drive two static opto-isolated relays to control the two normally closed solenoid valves (i.e. 2-2 for suction cup and 3-2 for pneumatic cylinder) connected to two pressure regulators. A compact Venturi effect system (i.e., piINLINE vacuum generator Micro Si by PIAB with a maximum vacuum pressure of −0.8 bar and a supply pressure of 6 bar) is used for the prototype to generate the vacuum in the suction cup. It is connected to the corresponding 3-2 solenoid valve (refer to Section 3 for the pneumatic schematics of both the vacuum system and the pneumatic cylinder).

The 256-K-I2C-CMOS-Serial-EEPROM-24LC256 (by Microchip) external memory is used to save the user and system configuration parameters, and to manage effects caused by power failures or controller shutdowns.

A pushbutton and an external input signals board, both equipped with opto-isolated static relays, are implemented to drive the gripper manually by the user or automatically through an external controller (e.g. a robot controller) via digital output signals.

The Ethernet Shield v.2 (by Arduino) enables the gripper to communicate via TCP/IP with an external control system (e.g. PC) to receive commands and send response messages.

The developed micro-gripper control framework comprises an embedded firmware layer and an external supervisory software layer, each addressing distinct control, management and supervision tasks. The MCU firmware handles all low-level, pseudo-real-time actuation and closed-loop control. Specifically, it executes trajectory generation and closed-loop PID regulation for finger positioning in friction grasping, two-state control of the vacuum system for vacuum grasping, and positioning of the pneumatic cylinder for the hybrid grasping mode. The sensors' acquisition and actuators' control logic are executed locally on the MCU to ensure deterministic and high-speed operation.

MATLAB is used strictly as an external management and supervisory tool. Indeed, MATLAB scripts send high-level commands to the MCU firmware to perform the desired operations with the micro-gripper and are employed to acquire, monitor, and analyse the micro-gripper state and motion data. This includes data logging for system identification and PID tuning of the finger linear axes. This architecture guarantees reliable embedded control on the MCU while leveraging MATLAB for operation management, performance analysis, supervision, and parameter optimisation.

### 2.3 Firmware and Software

As mentioned, a dedicated firmware has been developed to control the micro-gripper. This software implements a pseudo-real-time, multi-threaded control framework composed of three time-scheduled threads with different cycle times (refer to Fig. 5). The firmware enables the asynchronous management of actuators' control (i.e. the *CONTROL loop*), external communication (i.e. the *COM loop*), and manual/automatic operational modes (i.e. the *M/A MODE loop*), ensuring responsive and reliable gripper behaviour. The CONTROL loop implements the control algorithms for the fingers' positioning and two-state control of the vacuum supply for the suction cup and the vertical axis. The firmware supports three command input methods: manual control via pushbuttons, automatic control via external digital signals and Ethernet communication via TCP/IP. The TCP/IP communication remains active, while the selection between manual (i.e. pushbuttons) and automatic (i.e. external digital signals) operational modes is managed via a selector switch. These two operational modes are mutually exclusive.

**Fig. 5:**
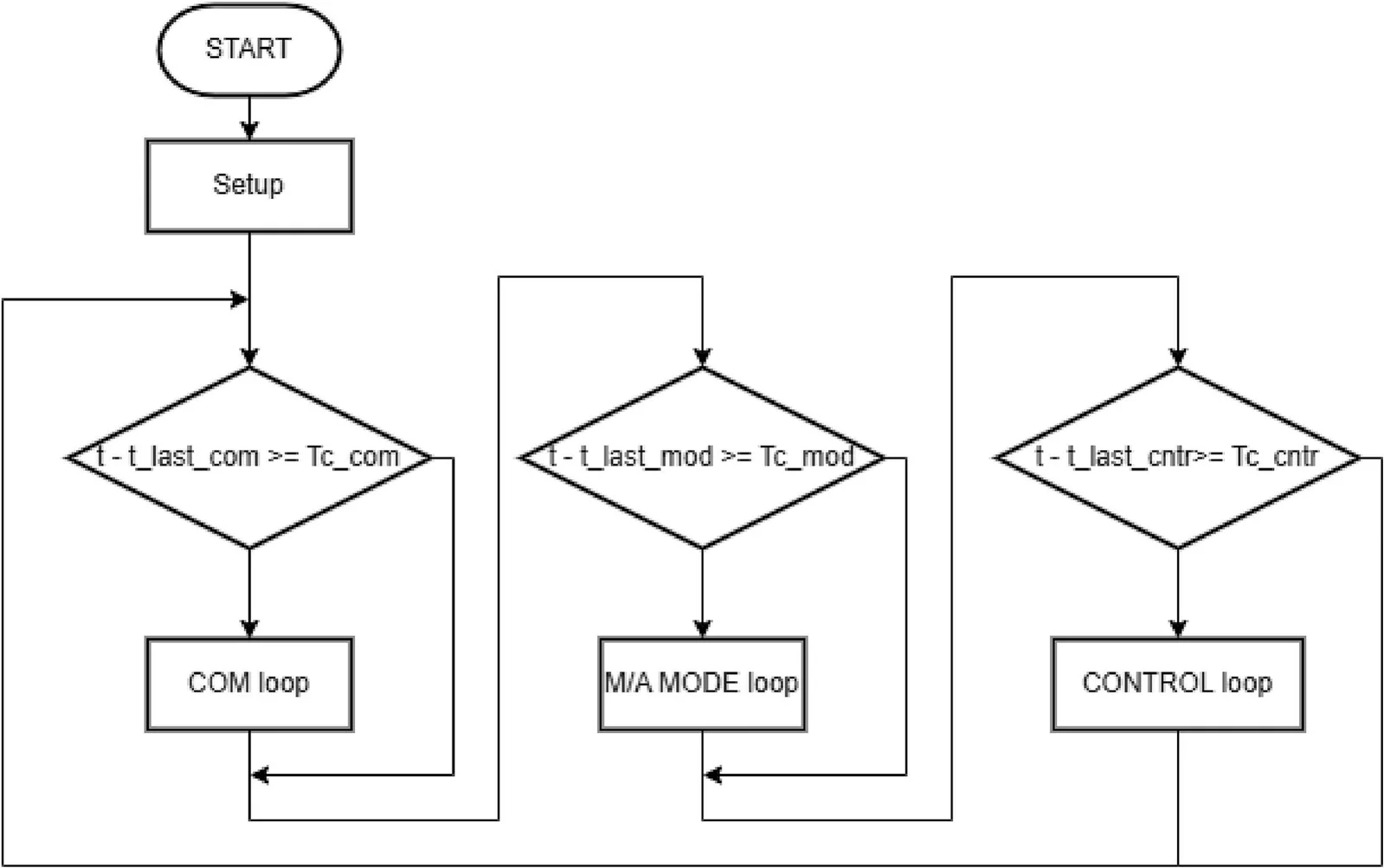
Flowchart of the micro gripper control software.

The COM loop manages TCP/IP communication with external systems (e.g. a PC) using a dedicated class that implements a state machine for handling client connections, establishing communication with recognised clients (i.e identified with trusted IP addresses), receiving commands to execute (e.g., requesting gripper status information) and sending response messages (e.g. a string with status info).

The implemented commands allow for calibration and control of finger motion, actuation of the pneumatic cylinder, control of the vacuum supply of the suction cup, system and user configuration parameter setting and system status monitoring.

A dedicated class manages the controllers of the linear stages, including the two-state controllers for the suction cup and the vertical axis. Moreover, this class manages the object dedicated to storing and retrieving the configuration parameters from external non-volatile memory (i.e. EEPROM).

During the initialisation of the controller object, all the peripherals of the MCU are properly configured. The fingers' controllers allow both open- and closed-loop motion control of the relative and absolute positioning. The implemented open-loop control algorithm is based on the method conceived in [46] for generating a trapezoidal/triangular speed profile for stepper motors to perform the desired movement. The motion profile was approximated with a sequence of discrete speed steps, calculated online, each with a specific duration, corresponding to the time interval between one motor step and the next. Instead, the designed closed-loop motion control was performed using a PID controller, with anti-windup compensation and feedforward action, optimised for trapezoidal motion law tracking. Based on the user position command (i.e. position setpoint) and configured parameters, the motion law is computed online, and the position reference (i.e. trajectory setpoint) is used as input for the control loop. The control variable (i.e. combined PID and feedforward actions) is the step frequency (i.e the stepping speed) that is used to generate a square wave (i.e. high-frequency timer) as input for the stepper motor driver. Position feedback was obtained using a highly accurate linear potentiometer. The designed algorithms are explained in more detail in [39].

#### 2.3.1 Software configuration

To ensure efficient gripper operations, it is necessary to set the configuration parameters and perform position sensor calibration and PID tuning. The system configuration parameters are organised into three separate files:

• *ConfigurationParameters.h*: contains the default values of parameters saved into the external non-volatile memory, regarding the status of the gripper, the motion profile parameters, the sensor calibration values, step size and control tolerance. Moreover, the memory addresses of the configuration parameters saved in the EEPROM (i.e. the structure of the used memory) are reported and explained in the file “ConfigurationParameters.h”.
• *GripperParameters.h*: includes the pin mapping of the electrical connections to the MCU, the configuration parameters for the ADC, the sensor calibration, the position feedback filtering, and both the PID and two-state controllers.
• *EthernetParameters.h*: defines the TCP/IP connection parameters, trusted client IP addresses, and the implemented command messages.

The following paragraphs explain how the identification tests were conducted.

#### 2.3.2 Linear step size estimation

Since the linear step size was initially unknown, it was experimentally estimated. A simple routine was conducted to perform the linear step size measurement. First, an open-loop command was executed with a relative movement sufficient to exceed the maximum mechanical backlash, in order to eliminate its effect and prevent inaccurate estimation of the linear step size. Therefore, a second movement was executed with a sufficient number of steps to minimise the uncertainty of the step size estimation. The distance covered by the last movement was measured using an absolute linear sensor with a linear resolution of 0.001 mm (i.e., Mitutoyo Digimatic Indicator ID-C112B No. 543-250B), and the estimation of the linear step size was obtained as the ratio between the travelled distance in micrometers and the number of commanded steps.

#### 2.3.3 Position sensor calibration

The software integrates an IIR filter with a cutoff frequency of 50 Hz to remove noise from the position feedback of potentiometers. The first step necessary to perform the sensor calibration is the verification of the maximum stroke of the linear stages, which were obtained using an absolute linear sensor (i.e., Mitutoyo Digimatic Indicator ID-C112B No. 543-250B) For each stage, the stroke was determined by measuring the linear displacement of the axis from the first limit switch, which represents the fully open position, to the second limit switch, representing the fully closed position. The calibration routine is performed by an automatic task implemented into the control firmware of the gripper (refer to Section 7.1 for a comprehensive description).

#### 2.3.4 System identification and PID tuning

A linear system identification test was first performed to fine-tune the PID controllers of the linear stages (refer to Section 7.1 for a comprehensive description). A step-response identification in the time domain was carried out for each linear stage, involving several open-loop motion cycles. During each motion cycle, a trapezoidal speed was set as the system input, and the output positions were acquired from the calibrated potentiometer, with a sampling time of 0.5 ms. Eight cycles were executed for each finger axis, varying the maximum motion speed from 3 mm/s to 24 mm/s and the motion time from 0.4 s to 3 s. From these trials, position data from six randomly chosen cycles were merged to estimate the first-order transfer function for each linear stage using the MATLAB System Identification toolbox. The validation of the system identification was then performed using the position data from the remaining two cycles. This process ensured accurate system dynamics modelling, facilitating the fine-tuning of the PID controllers. The performed system identification can be found in the MATLAB live script “Identification.mlx”.

The fine-tuning of the PID controllers was performed using the MATLAB PID Tuner toolbox. A PI controller was chosen for both linear stages, with the robustness set to the maximum value to avoid unwanted overshoots and to ensure an optimised trade-off between setpoint tracking and disturbance rejection.

#### 2.3.5 Software for data acquisition and logging (MATLAB programs)

A MATLAB program was developed to allow motion data logging for position sensor calibration, system identification, and debugging during gripper working operations. This program continuously acquires position feedback, motor control frequency, trajectory setpoint, and time data for the linear stage of both fingers. A serial communication has been configured for data acquisition. The MATLAB script “SerialAcquisition.m” initiates the connection with the MCU via the serial port (i.e. constant variable “SERIAL_PORT”), with a baud rate (i.e. constant variable “BAUDRATE”) of 115200. The script creates an online 2D plot in the time domain using the received data and retains a number of samples defined by a user-defined constant variable (i.e. “N_MAX_ACQ”). Communication is interrupted when the Enter key on the keyboard is pressed. Finally, the data are saved from the MATLAB workspace. To allow the MCU to send finger motion data, the system configuration constant variable “DEBUG_DATA_SEND” in the gripper control framework must be set to 1; if set to 0, the serial communication will not be enabled.

As previously mentioned, the gripper operational commands and the corresponding responses are transmitted via Ethernet communication, adopting the TCP/IP protocol. Existing software such as Packet Sender, Hercules, or MATLAB can be used to communicate with the gripper.

#### 2.3.6 Ethernet communication protocol

To establish Ethernet communication with the gripper, the network interface of the external control system (e.g., a PC) must be configured with a static IP address, which is included in the list of recognised clients defined in the configuration parameters of the gripper control framework.

The communication messages are contained between the ASCII symbols “<” and “>”, and all the messages must be terminated with the ASCII terminator character (i.e. the carriage return).

During the connection, when a client with a trusted IP address is recognised by the MCU (i.e., the server), it must send the handshake string (i.e., “<HANDSHAKE>”) followed by the terminator character. The server responds with an acknowledgement message (i.e., “<ACK>”), completing the connection procedure. Otherwise, if the handshake message is missing or incorrect (or if command messages are wrong), the server returns an error message (i.e. “<ERR>”). Moreover, if the IP address is not recognised, the MCU rejects the connection and continues waiting for a verified client.

Once the connection is established, the client can send the commands reported in the “EthernetConfiguration.h” file and receive the corresponding response messages. Moreover, the defined syntax and semantics of the TCP/IP communication protocol (i.e. the list of available commands and their corresponding responses, including detailed explanations) are also provided in the file “Ethernet commands.xlsx” supplied with the firmware and software (refer to Section 3).

The connected client can terminate the Ethernet communication with the MCU by sending a termination message (i.e. “<STOP>”), after which the server will again wait for new connections.

## 3 Design files summary

This section reports all the manufactured parts. Most of them are produced by additive manufacturing, while cutting technologies were employed only for components requiring high mechanical resistance and high-precision dimensional and geometric tolerances.

The whole design is done by Autodesk Inventor Professional 2022, and the original file (i.e. .ipt) is supplied for each part. Additionally, the STEP files (i.e. .stp) are generated for every part and for the 3D printed manufactured parts, the STL files (i.e. .stl) are attached.

Sub-assemblies and the micro-gripper assembly are provided in the original Autodesk Inventor format (.iam) and as STEP files. All the design files are listed in Table 1, and the related parts and assemblies are shown in Fig. 6, Fig. 7.

**Table 1:** Design File Summary Table.

**Design file name**	**File type**	**Open source license**	**Location of the file**
MobileFrame15	CAD Part File	CC BY 4.0	
Pin3-30	CAD Part File	CC BY 4.0	https://doi.org/10.17632/bpsmdb9nkf.1
CentralPart12	CAD Part File	CC BY 4.0	https://doi.org/10.17632/bpsmdb9nkf.1
PotPlate2	CAD Part File	CC BY 4.0	https://doi.org/10.17632/bpsmdb9nkf.1
PotCon1	CAD Part File	CC BY 4.0	https://doi.org/10.17632/bpsmdb9nkf.1
Nail8L	CAD Part File	CC BY 4.0	https://doi.org/10.17632/bpsmdb9nkf.1
PistonVert2	CAD Part File	CC BY 4.0	https://doi.org/10.17632/bpsmdb9nkf.1
CylVert2	CAD Part File	CC BY 4.0	https://doi.org/10.17632/bpsmdb9nkf.1
Base9	CAD Part File	CC BY 4.0	https://doi.org/10.17632/bpsmdb9nkf.1
Flange2	CAD Part File	CC BY 4.0	https://doi.org/10.17632/bpsmdb9nkf.1
MobileFrame15_A	CAD Assembly File	CC BY 4.0	https://doi.org/10.17632/bpsmdb9nkf.1
Base9_A	CAD Assembly File	CC BY 4.0	https://doi.org/10.17632/bpsmdb9nkf.1
CentralPart12_A	CAD Assembly File	CC BY 4.0	https://doi.org/10.17632/bpsmdb9nkf.1
CylVert5Base_A	CAD Assembly File	CC BY 4.0	https://doi.org/10.17632/bpsmdb9nkf.1
Gripper15	CAD Assembly File	CC BY 4.0	https://doi.org/10.17632/bpsmdb9nkf.1
Electronic Schematic	PDF	CC BY 4.0	https://doi.org/10.17632/bpsmdb9nkf.1
Pneumatic Schematic	PDF	CC BY 4.0	https://doi.org/10.17632/bpsmdb9nkf.1
Firmware and Software	Arduino and MATLAB programs	CC BY 4.0	https://doi.org/10.17632/bpsmdb9nkf.1

**Fig. 6:**
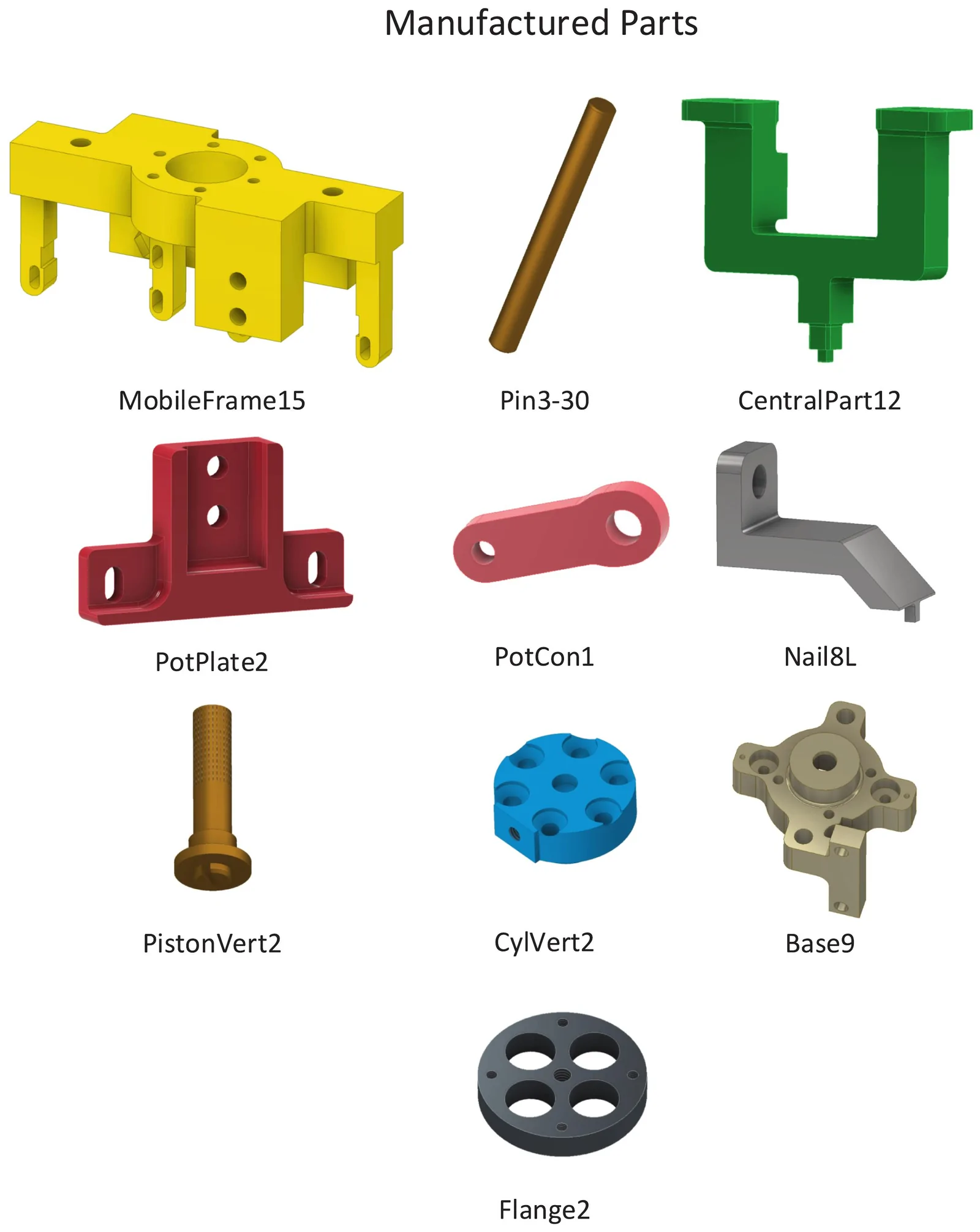
3D representation of the micro-gripper mechanical components (not to the same scale).

**Fig. 7:**
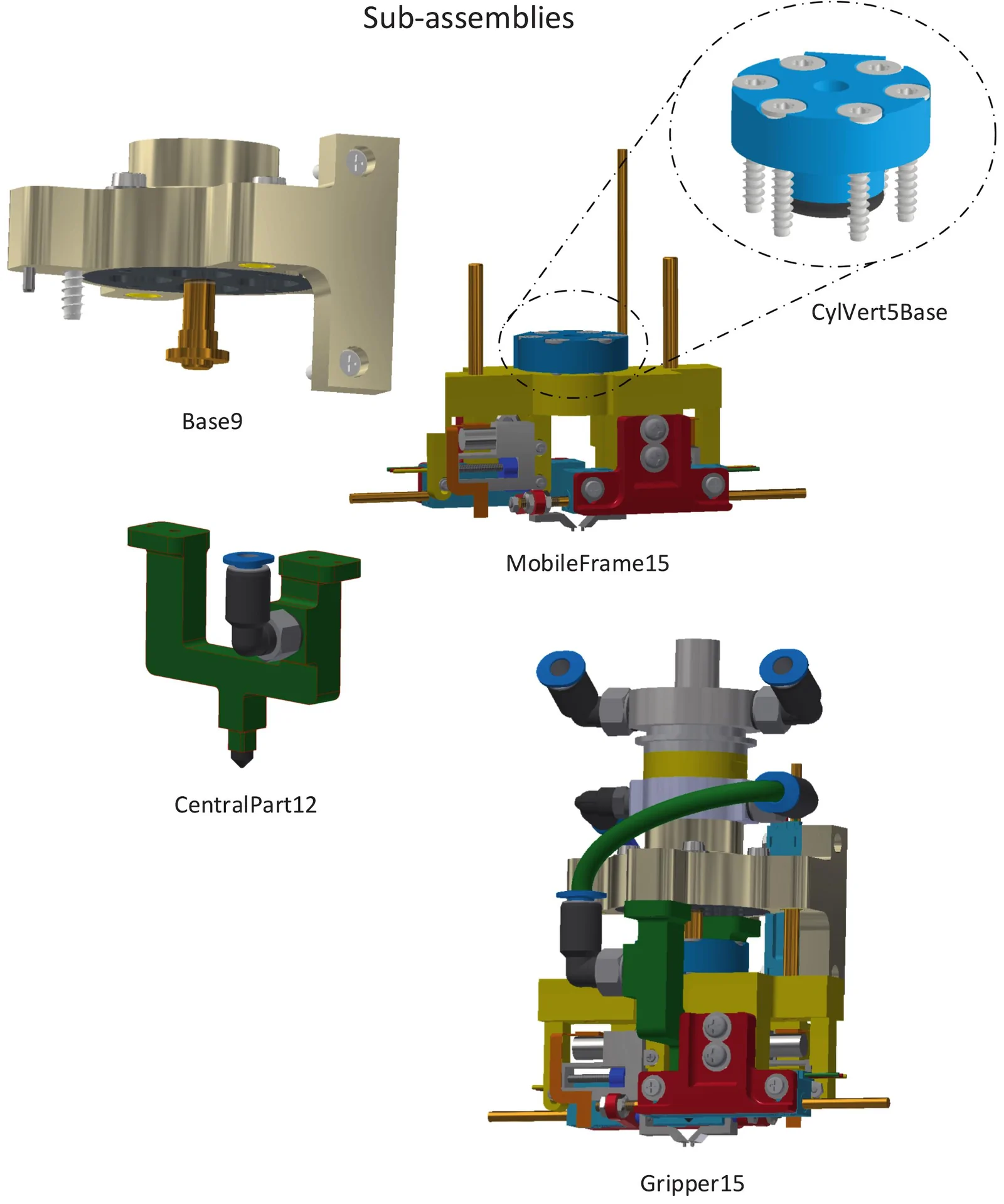
3D representation of the micro-gripper and its sub-assemblies (not to the same scale).

A brief description of all the design files included in the project is provided below:

*MobileFrame 15*

It is a 3D-printed part that serves as the holder for the friction-grasping tool. It houses the potentiometers, miniature linear stages, and fingers.

*Pin3-30:*

Brass pins act as guide shafts, allowing the MobileFrame15 to move smoothly up and down relative to the Base9.

*CentralPart12*

It is a 3D-printed part that serves as the holder for the vacuum grasping tool. It houses the suction cup and the pneumatic fitting for the vacuum supply.

*PotPlate2*

They are 3D-printed parts that mount the potentiometers of the fingers to the MobileFrame15.

*PotCon1*

They are 3D-printed parts that connect the miniature linear stage’s nut-driven carriage to the potentiometer's guided push rod for each finger.

*Nail8L*

They are the 3D-printed L-shaped fingers of the friction grasping tool. They can be customised and replaced according to the specific applications.

*PistonVert2*

It is the metal piston rod of the pneumatic cylinder, manufactured using cutting technology. It enables the MobileFrame15 to move vertically.

*CylVert2*

It is the 3D-printed cylinder of the piston rod (i.e. PistonVert2). It houses the pneumatic fitting for the compressed air supply.

*Base9*

It is a 3D-printed part that serves as the fixed base of the micro-gripper. It is the top part of the micro-gripper, and it integrates the mechanical interface where the automatic tool changer (or a fixed interface) of the robot is mounted.

*Flange2*

The metal interface, manufactured using cutting technology, attaches the PistonVert2 to the Base9. This interface is screwed in the Base9.

*Mobile Frame15_A*

It is the sub-assembly of the friction grasping tool with fingers, linear stages, and potentiometers.

Base9_A

It is the sub-assembly Base for the MobileFrame15, with the piston rod of the cylinder.

*CentralPart12_A*

It is the sub-assembly of the vacuum grasping tool with the suction cup

*CylVert5Base_A*

It is the sub-assembly of the pneumatic cylinder.

*Gripper15*

It is the manufactured and assembled prototype of the multi-mode hybrid micro-gripper.

*Pneumatic Schematic*

It is the pneumatic schematic of the gripper for the vacuum supply to the suction cup and the pneumatic cylinder.

*Electronic Schematics*

It is the electronic schematic of the gripper control system.

*Firmware and software*

The Arduino and MATLAB programs allow the gripper to operate. The control system framework, called “HybridMicroGripperController”, is inside the Arduino folder. The MATLAB software, used for acquiring and plotting data, identifying the system and tuning the PID controller, is inside the MATLAB subfolder.

Fig. 6 shows the manufactured parts of the designed micro-gripper, while Fig. 7 represents the micro-gripper (Gripper15) with its sub-assemblies. It is worth to note that the micro-gripper is assembled around its fixed part (Base9). This fixed part integrates the friction grasping (MobileFrame15 – mobile tool that can move vertically) and the vacuum grasping (CentralPart12 – fixed tool) tools.

## 4 Bill of Materials (BoM) summary.

Table 2 reports all the parts and materials used for manufacturing the micro-gripper prototype.

**Table 2:** Bill of Materials Table.

**Designator**	**Component**	**Number**	**Cost per unit −currency**	**Total cost −** **currency**	**Source of materials**	**Material type**
1	MobileFrame15	1				3D-Printed Polymer
2	Pin3-30	2				Metal
3	Microstage1	2	1.00 €	2.00 €	https://www.aliexpress.com/	Other
4	SNZS-M1.4-6	6		11.45 € (50 pcs)	https://it.misumi-ec.com	Other
5	HNT1-SUS-DIN1.4	6	1.20 €	7.20 €	https://it.misumi-ec.com	Other
6	CentralPart12	1				3D-Printed Polymer
7	QSML-M5-4	1		37.20 € (10 pcs)	https://it.rs-online.com	Other
8	R7M-0050-1	1	10.00 €	10.00 €	https://www.micro-mechanics.com/	Rubber
9	MM10-8	2	48.00 €	96.00 €	https://www.megatron.de	Other
10	PotPlate2	2				3D-Printed Polymer
11	ISO 7089-2.5	4				Other
12	NKJ2.5-10	4	1.02 €	4.08 €	https://it.misumi-ec.com	Other
12a	ISO4032-M2.5	4				Other
13	ISO 7089-2	4				Other
14	CSPPN3P-STN-M2-12	4	1.98 €	7.92 €	https://it.misumi-ec.com	Other
14a	ISO4032-M2	4				Other
15	ISO4032-M2	4				Other
16	PotCon1	2				3D-Printed Polymer
17	Nail8L	1				3D-Printed Polymer
18	HNT1-SUS-DIN-M1.4	2	1.20 €	2.40 €	https://it.misumi-ec.com	Other
19	SNZS-M1.4-6	6		11.45 € (50 pcs)	https://it.misumi-ec.com	Other
20	PistonVert2	1				Metal (Machined)
21	CylVert2	1				3D-Printed Polymer
22	STP410250150	6	1.21 €	7.26 €	https://de.screwerk.com	Other
23	OR-8x2	1		5.58 € (100 pcs)	https://it.rs-online.com	Elastomer
24	Base9	1				3D-Printed Polymer
25	ISO2338 – 1.5h8x12	2				Other
26	STP380300120	2	1.21 €	2.42 €	https://de.screwerk.com	Other
27	ISO1207 M2.5x10	4				Other
28	ISO 7089-2.5	4				Other
29	GSM-0304-05-02	2	2.17	4.34 €	https://it.misumi-ec.com	Other
30	MM10-8	1	48.00 €	48.00 €	https://www.megatron.de	Other
31	CSPPN3P-STN-M2-12	2	1.98	3.96 €	https://it.misumi-ec.com	Other
32	ISO 4032 M2	2			smallware	Other
33	ISO 4032 M6	1			smallware	Other
34	Flange2	1				Metal (Machined)
35	ISO 4032 M4	1			smallware	Other
37	Robot Interface	1				Metal (Machined)
38	QSML-M5-4	1	3.72 €	3.72 €	https://it.rs-online.com	Other
39	QSML-B-M3-3	1	5.36 €	5.36 €	https://it.rs-online.com	Other
40	SCHUNK-MWA-20-1 Adapter	1	250.00 €	250.00 €	https://schunk.com/it	Other
41	Pneumatic Hose ø 4	100 mm			https://it.rs-online.com	Polymer
42	Pneumatic Hose ø 3	100 mm			https://it.rs-online.com	Polymer
43	SCHUNK-MWK-20-1 Head	1	500.00 €	500.00 €	https://schunk.com/it	Other
44	QSML-M5-4	2	3.72 €	7.44 €	https://it.rs-online.com	Other
XA1	Arduino Ethernet Shield 2	1	25.80 €	25.80 €	https://store.arduino.cc/products/arduino-ethernet-shield-2	Semiconductor
XA2	Arduino Due	1	42.00 €	42.00 €	https://store.arduino.cc/products/arduino-due	Semiconductor
K1, K4, K5, K6, K7, K8	AQY210EH PhotoMOS	6	2.6 €	15.60 €	https://co-en.rs-online.com/product/panasonic-electronic-components/aqy210eh/70158341/	Semiconductor
K2, K3	Relay module 5V 2 channels	1	3.50 €	3.50 €	https://www.amazon.it/dp/B01H2D2RI0	Semiconductor
U1, U2	STSPIN220 Low-Voltage Stepper Motor Driver Carrier	2	7.95 €	15.90 €	https://www.pololu.com/product/2876	Semiconductor
U3	24LC256 EEPROM	1	1.04 €	2.08 €	https://www.microchip.com/en-us/product/24lc256#purchase-from-store	Semiconductor
**Priced items**All priced items, excluding 40, 43				382.66 €		
**Blank-cost items**Upper bounds of estimated cost: smallware 20 €, 3D-printed 80 €, machined 80 €				180.00 €		
**TOTAL**Without tool changer				**562.66 €**		
**Tool changer**Items 40, 43 (optional)				750.00 €		

Legend:

The blank unit-cost, total-cost, and supplier fields refer to smallware, to 3D printed components, and to custom-manufactured components, as explained in the rows below;

Some components have different designators (i.e., numbers) even if they are the same part; e.g., component “4” is identical to “19”, and “14a” is the same as “15”. This duplication occurs because designators of the components were assigned sequentially during the gripper assembly process. As a result, identical components used in different assembly steps were given new designators, even if already present in the BoM;

The components identified as “ISO” are standard mechanical components; therefore, no additional information is needed;

The cost of smallware (i.e., blank fields in the table) is neglected. It is estimated to be approximately 10.00 ÷ 20.00 €;

The total estimated cost of all additive-manufactured parts (blank fields in the table) is approximately 60.00 ÷ 80.00 €;

All 3D printed components were realised by the 3D printer “Formlabs Form 3+” with the “Grey” resin. For the Nail8L (i.e., component “17”), a special “High Temp” resin was utilised.

All the parts indicated as “Metal (Machined)” are manufactured using cutting technology in a mechanical workshop equipped with CNC tools. The total estimated cost of all machined metal parts (blank fields in the table) is approximately 50.00 ÷ 80.00 €;

The automatic tool changer, composed of components “40” and “43”, is not strictly part of the micro-gripper design. The total cost of the tool changer used in this project is 750 €. Alternative tool changers or fixed interfaces, typically selected or designed depending on the robot flange, can be used.

The total cost (562.66 €) includes upper-bound estimates for the blank-cost items; it is therefore an overestimation.

The first column, “Designator”, reflects the numbering used in Section 5 (i.e. Build Instructions) for the mechanical components of the gripper. At the same time, it represents the code used in the electronic schematic of the gripper control system for electronic components.

## 5 Build instructions

This section illustrates and explains the steps to build the micro-gripper. Each step is described in detail using an exploded view. Components are added step by step and are identified by number. The numbers refer to the component designators assigned in the BoM (refer to Section 4).

**Step 1 – Assembly of the two vertical brass pins – (** Fig. 8 **)**

**Fig. 8:**
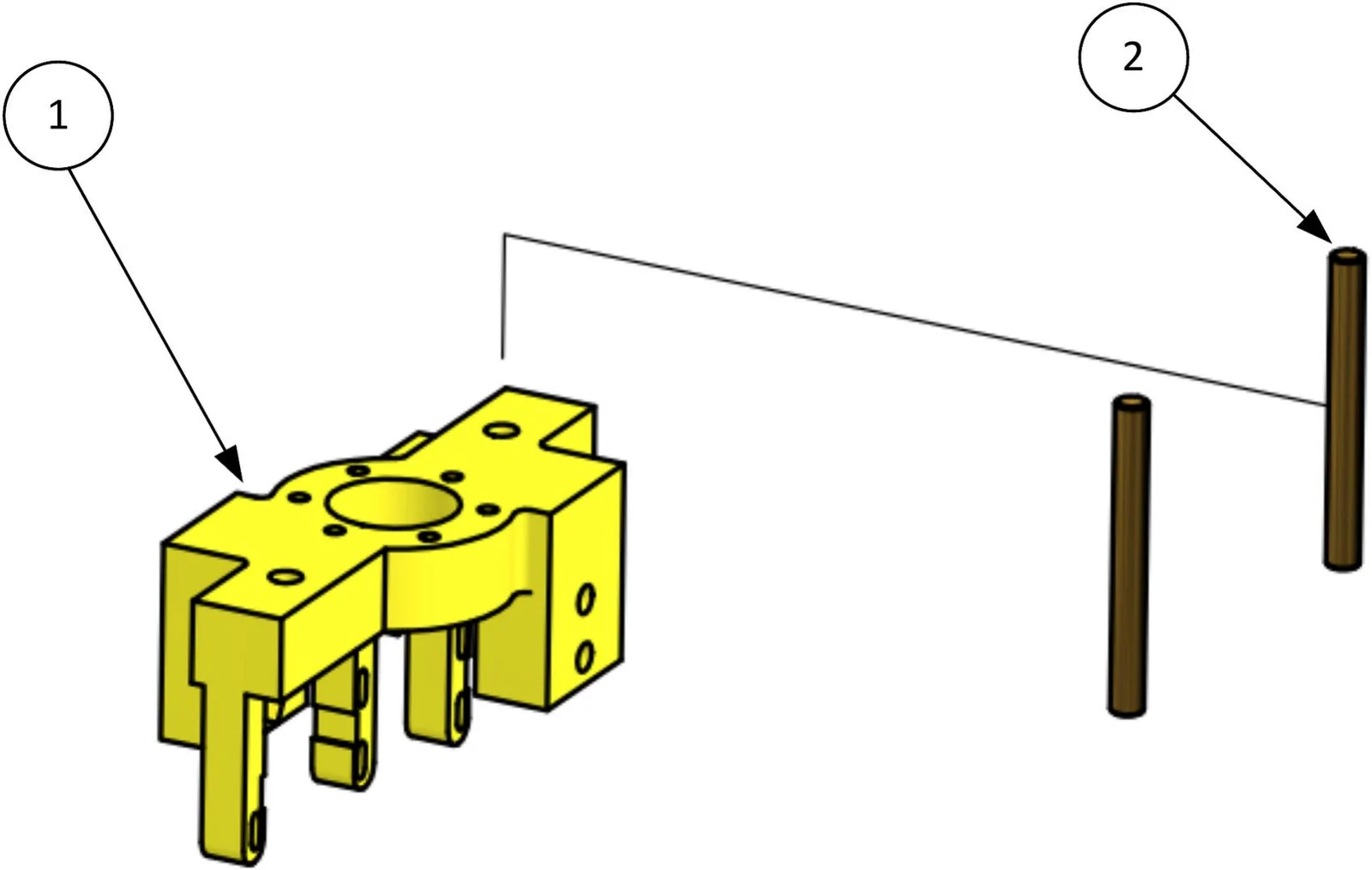
Vertical insertion of the two brass pins in the corresponding holes of the MobileFrame15.

The two brass bars (i.e. components “2”) are inserted vertically inside the corresponding holes in the MobileFrame15 (i.e. component “1”). Ensure the orthogonality between the vertical pins and the top surface of the MobileFrame15; to help with assembly, a set square can be used. Additionally, ensure the parallelism between the vertical pins. After the insertion, if the coupling is not an interference fit, the vertical pins can be glued in place using a cyanoacrylate adhesive.

**Step 2 – Assembly of the two miniature linear stages – (** Fig. 9 **)**

**Fig. 9:**
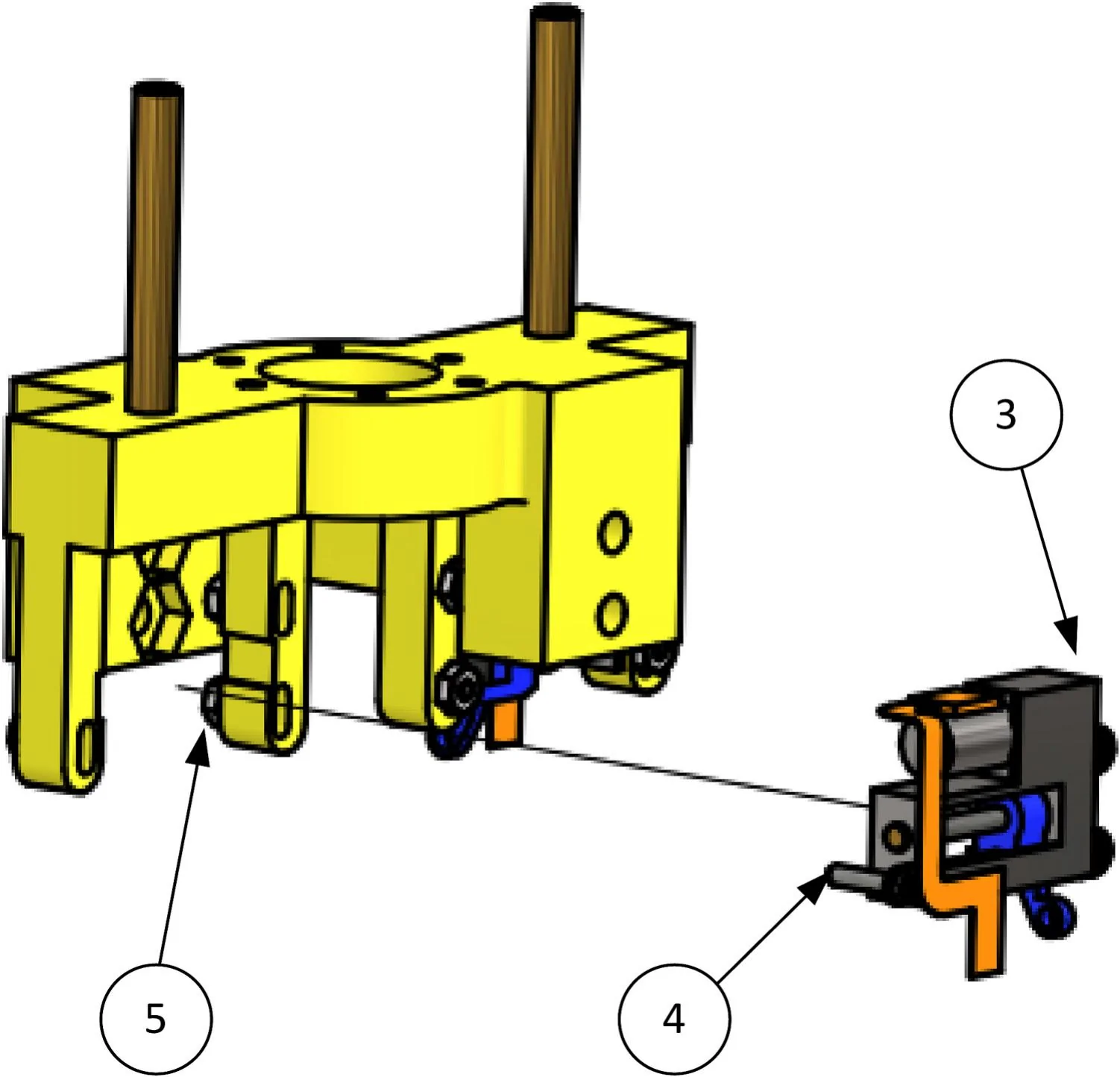
Assembly of the miniature linear stages on the MobileFrame15.

The two miniature linear stages (“3”) are assembled on the MobileFrame15 (i.e. yellow part in Fig. 9). Each linear stage is connected by three M1.4 screws (“4”) and nuts (“5”). The MobileFrame15 has four slots to align the two stages along the horizontal axis (i.e. two slots for each stage). At the end of this assembly step, the two holes of the nut-driven carriages (i.e. the blue parts) of the linear stages must be aligned along the horizontal axis.

**Step 3 – Central Part assembly– (** Fig. 10 **)**

**Fig. 10:**
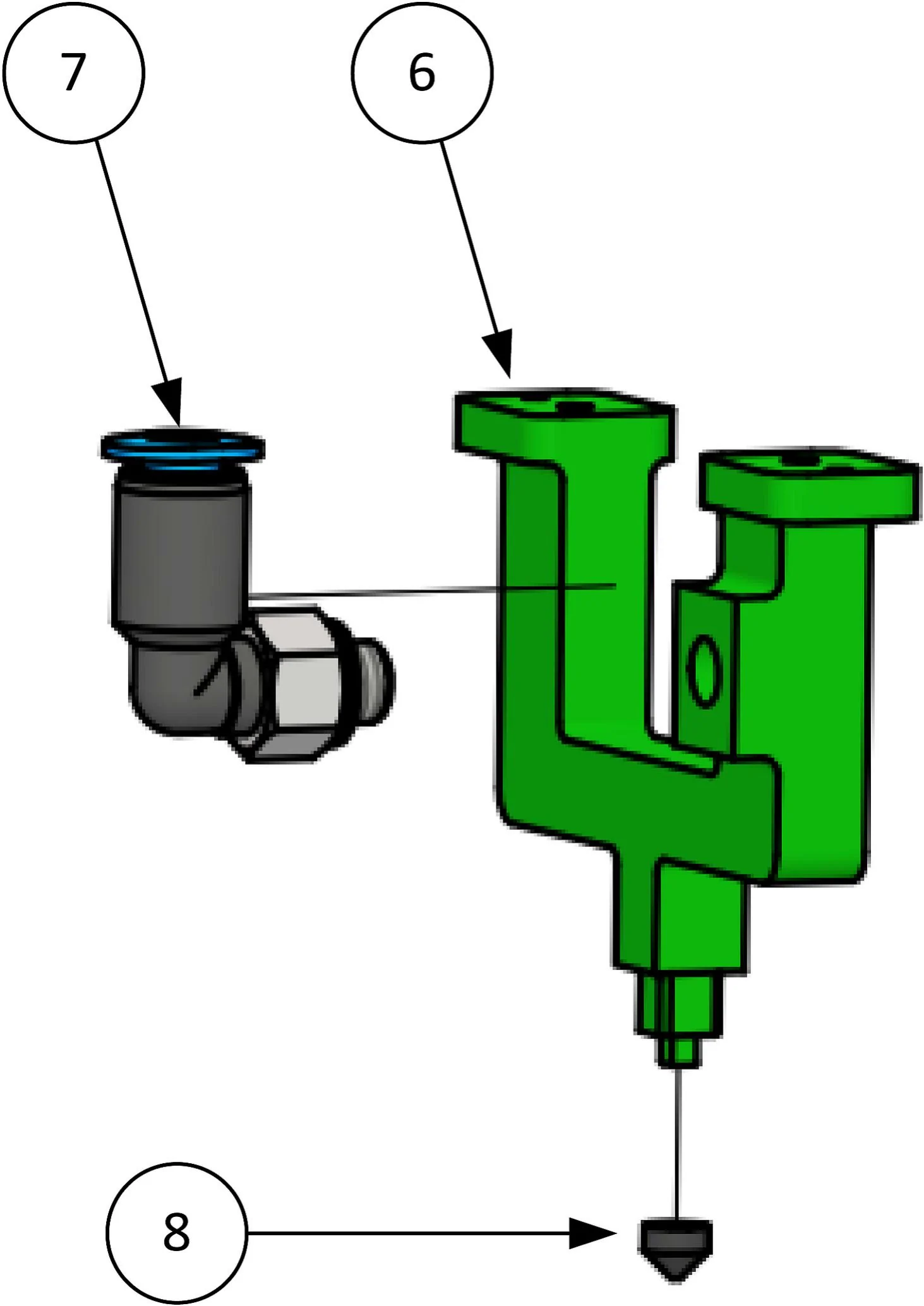
Assembly of the CentralPart12.

After assembling the MobileFrame15, it is necessary to mount the CentralPart12 sub-assembly. It is composed of a 3D-printed holder (“6”) with a push-in L pneumatic fitting (“7”) and a micro-suction cup (“8”).

The pneumatic fitting is screwed into the holder via a male M5 thread. The female M5 thread must be done into the 3D printed part since it is not manufactured with the thread. It is recommended to glue the M5 thread of the pneumatic fitting with a cyanoacrylate adhesive to prevent air leakage. It is also possible to use a metal threaded insert to obtain a reinforced female thread. The micro suction cup is mounted, pushing it into the holder.

**Step 4 – Central Part and Mobile Frame Assembly – (** Fig. 11 **)**

**Fig. 11:**
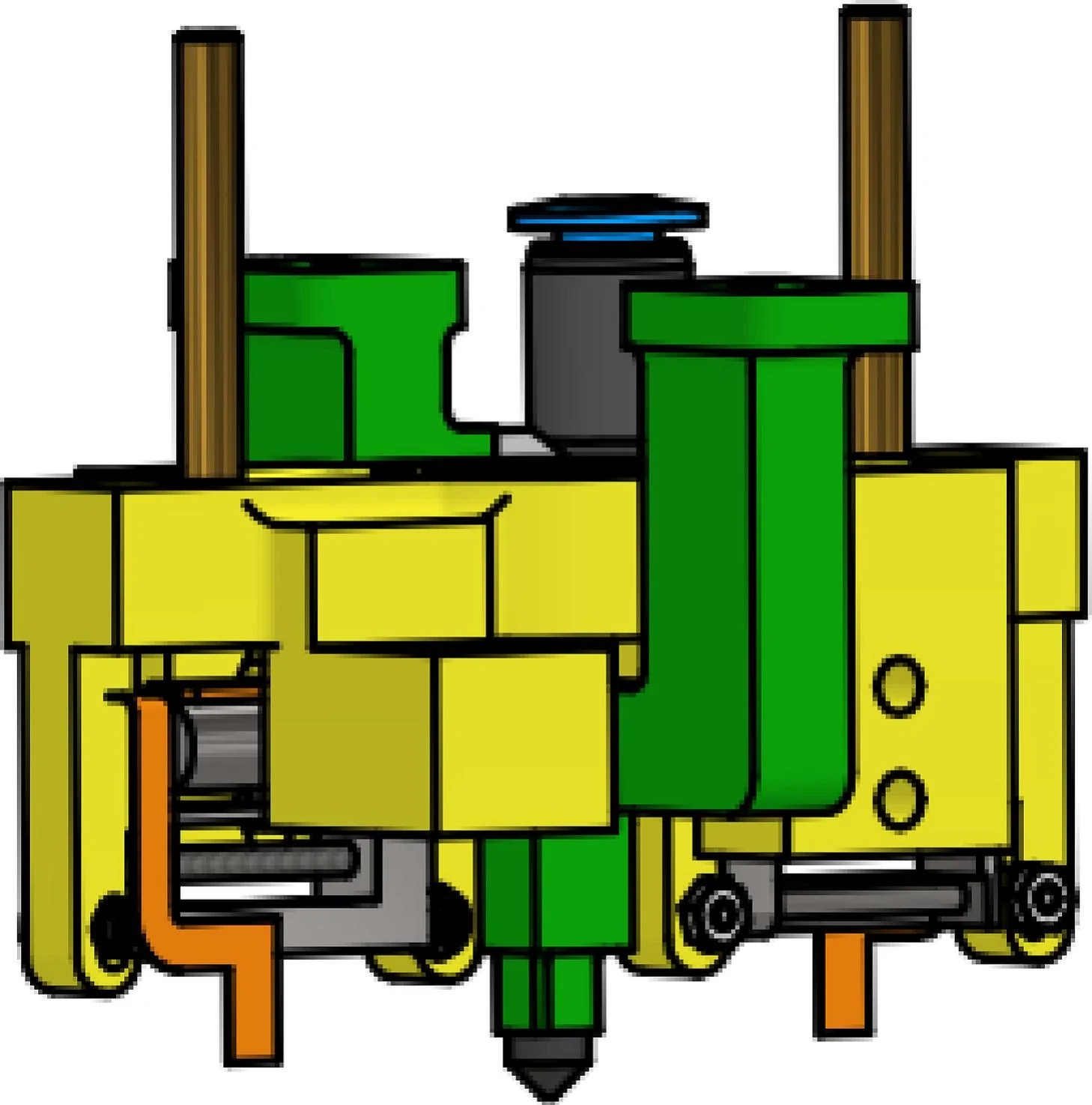
Joining the CentralPart12 and MobileFrame15 sub-assemblies.

Up to this point, the MobileFrame15 (i.e. yellow part in Fig. 11) and the CentralPart12 (i.e. green part) sub-assemblies have been assembled separately. In this step, these two parts are joined into a single sub-assembly, as shown in Fig. 11.

**Step 5 – Assembly of the linear stage potentiometers – (** Fig. 12 **)**

**Fig. 12:**
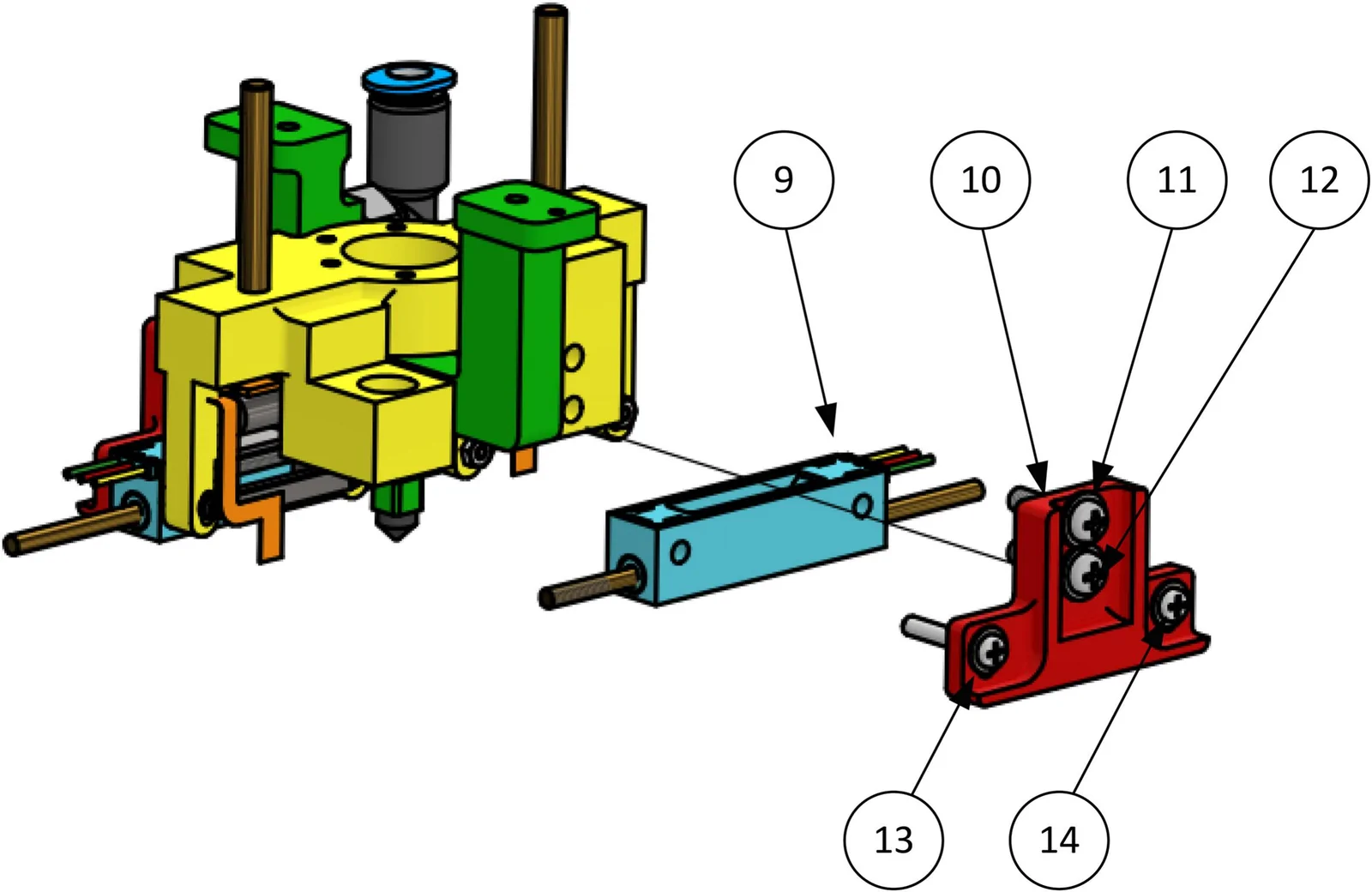
Assembly of the potentiometer (one for each micro linear stage).

The linear potentiometer of the miniature linear stages (“9”) is mounted on the MobileFrame15 (i.e. yellow part in Fig. 12) using the PotPlate2 (“10”), according to the following procedure:

• first fasten the potentiometer to PotPlate2 using the screws (“14”), washers (“13”) and nut
• s (“14a”, not shown in Fig. 12 since hidden by the potentiometer);
• next attach the resulting sub-assembly to the MobileFrame15 using screws (“12”), washers (“11”) and nuts (“12a”, not shown in Fig. 12 since hidden by the MobileFrame15)

This procedure must be carried out twice, once for each potentiometer.

**Design variant:**

Potentiometers are not mandatory for the working operations of the micro-gripper; however, their presence enables improved finger positioning accuracy by allowing closed-loop motion control. Otherwise, the micro-gripper fingers can be controlled only by adopting an open-loop motion strategy.

**Step 6 – Assembly of the fingers – (** Fig. 13 **)**

**Fig. 13:**
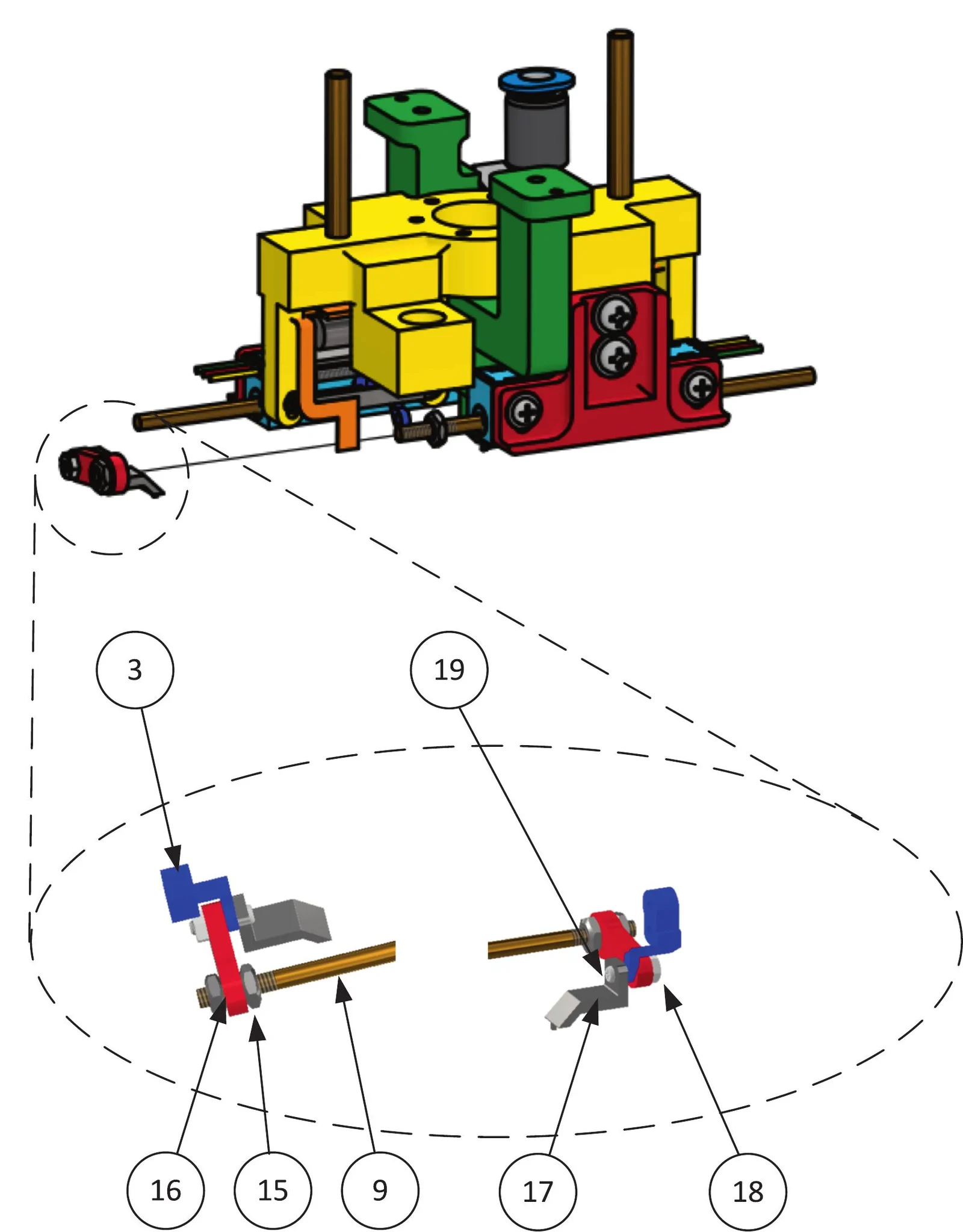
Details of the finger assembly.

This step consists of assembling the friction grasping fingers onto the miniature linear stages and connecting them to the potentiometers.

The PotCon1 (“16”) is a 3D printed part that connects the nut-driven carriage of each linear stage (“3”) to its corresponding guided push rod of the potentiometer (“9”). It is fastened with two nuts (“15”) to the potentiometer push rod.

Each finger (“17”) is connected to the corresponding nut-driven carriage of the linear stage and PotCon1 using the screw (“19”) and nut (“18”). Each couple of bolts (i.e., composed of the screws and nuts) and PotCon1 act as a mechanical linkage between the finger, the linear potentiometer, and the linear stage. This procedure must be carried out twice, once for each finger.

**Design variant:**

The design of fingers can be tailored for specific SMT component assembly applications. They can be designed for general-purpose uses, like those developed in this project, or adapted for particular component dimensions and geometries.

**Step 7 – Piston rod Insertion – (** Fig. 14 **)**

**Fig. 14:**
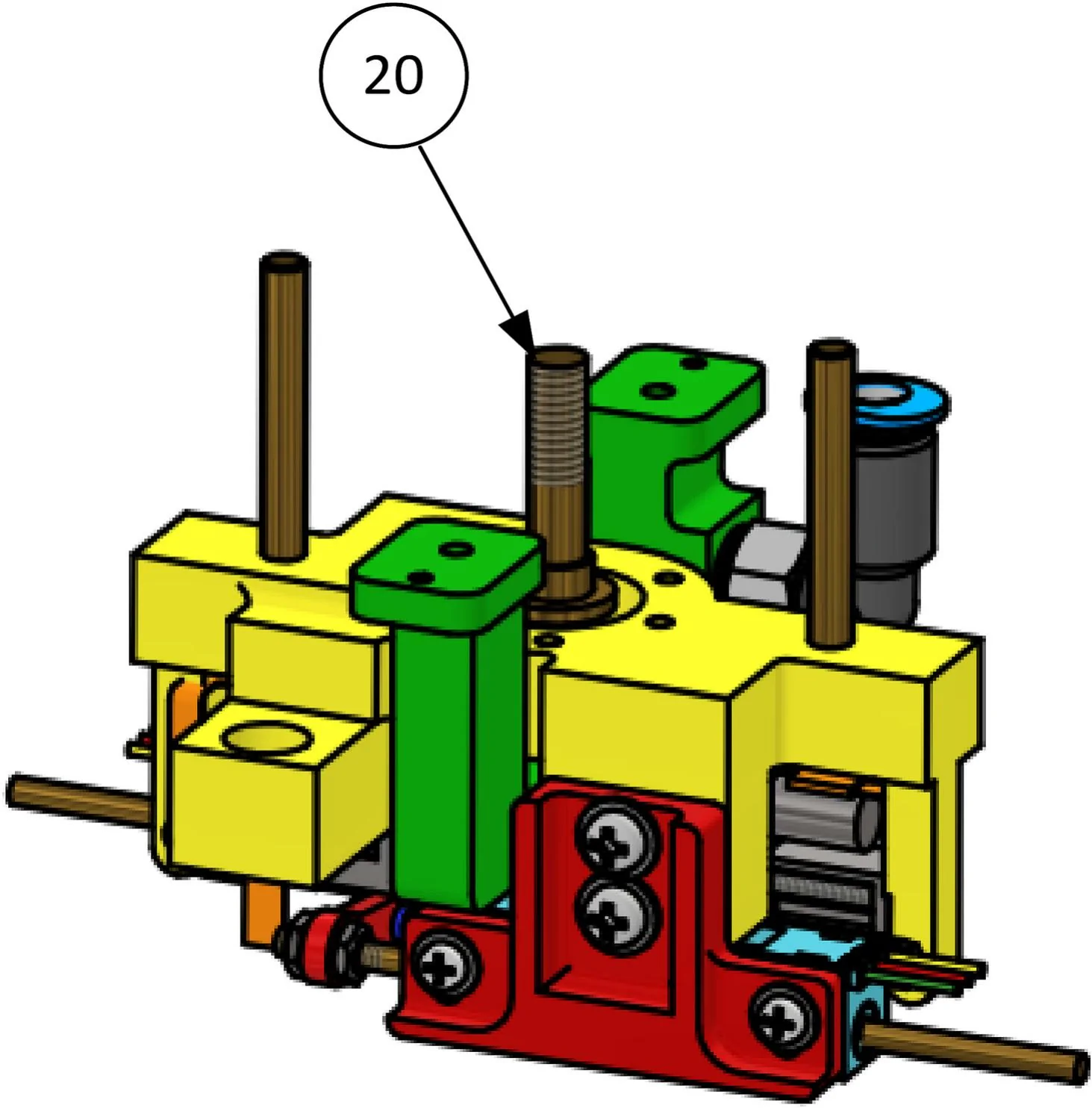
Piston rod insertion inside its housing.

In this step, the piston rod (“20”) of the pneumatic cylinder is mounted in its final position within the central hole on the top surface of MobileFrame15 (i.e. yellow part in Fig. 14).

**Step 8 – Pneumatic cylinder assembly – (** Fig. 15 **)**

**Fig. 15:**
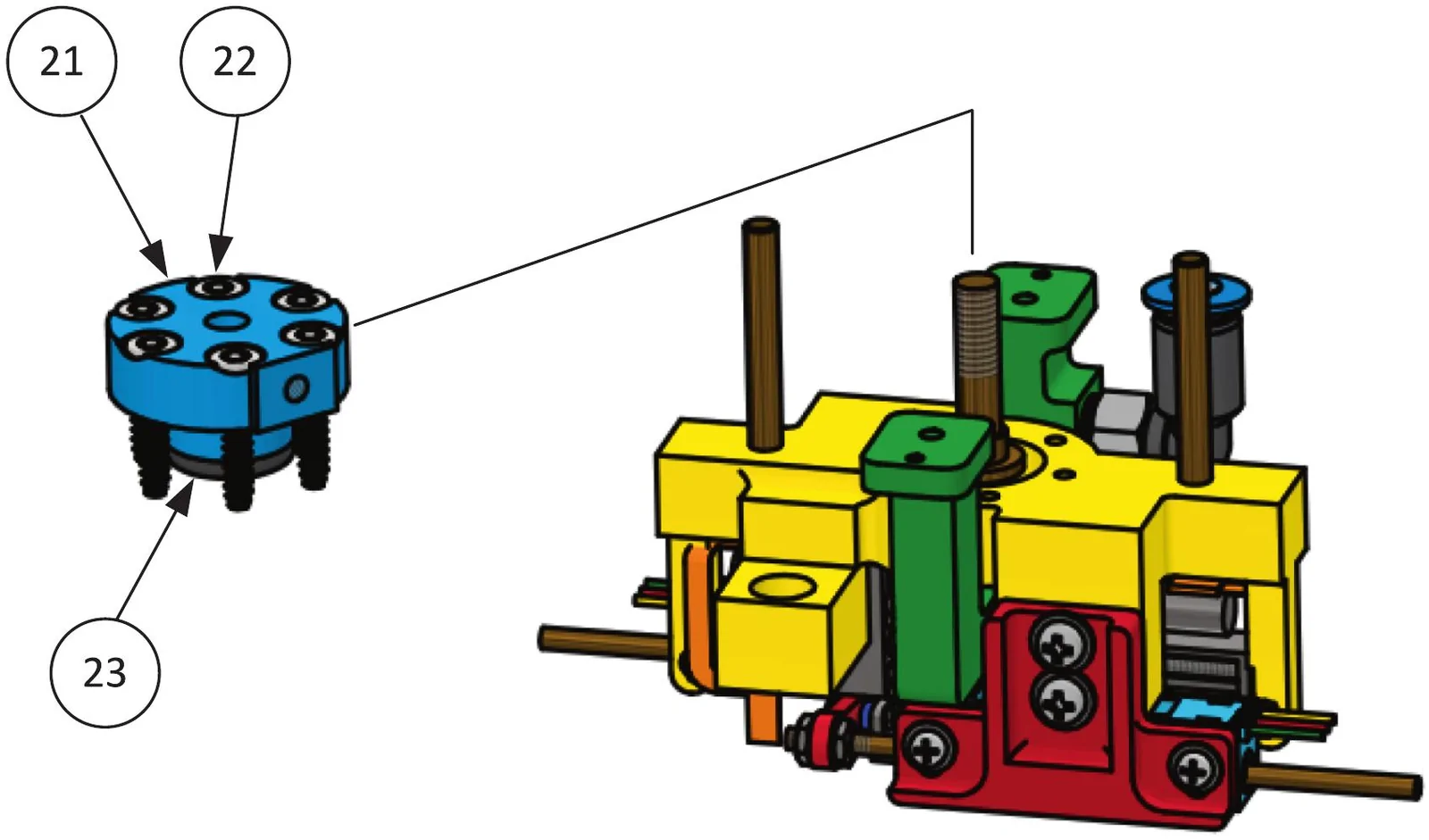
Pneumatic cylinder assembly.

The cylinder (“21”) is attached to MobileFrame15 (i.e., the yellow part in Fig. 15) using six screws (“22”), which are specifically designed for polymeric material. The push-in pneumatic fitting to supply compressed air is screwed into the cylinder via a male M4 thread. Moreover, an O-ring (“23”) is placed on the bottom surface of the cylinder.

**Step9 – Assembly of Flange2 – (** Fig. 16 **)**

**Fig. 16:**
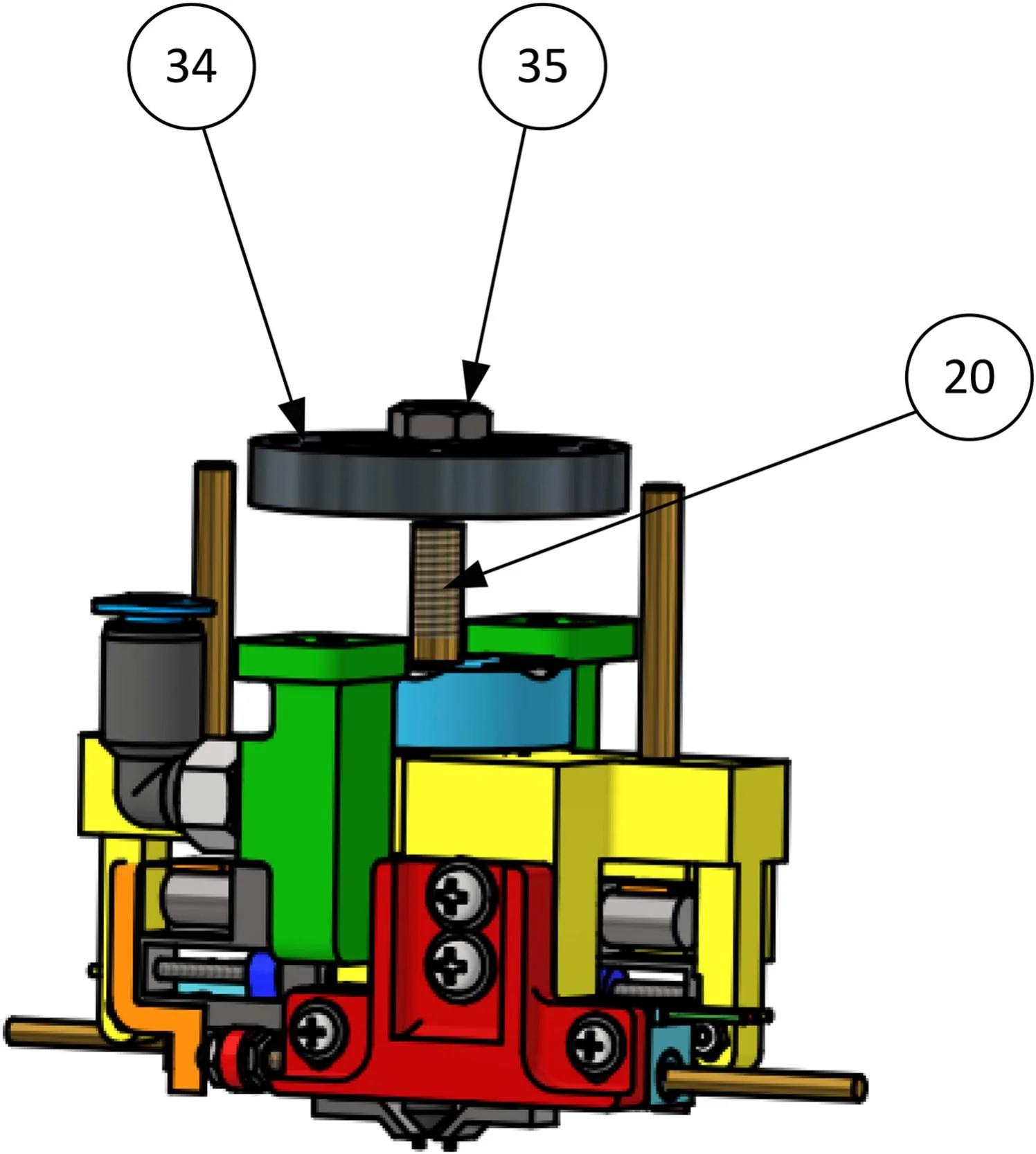
Assembly of Flange2 onto PistonVert2 (i.e. the piston rod).

In this step, the Flange2 (“34”) is screwed onto the PistonVert2 (“20”) using the locknut (“35”).

**Step 10 – Assembly of the pneumatic cylinder potentiometer – (** Fig. 17 **)**

**Fig. 17:**
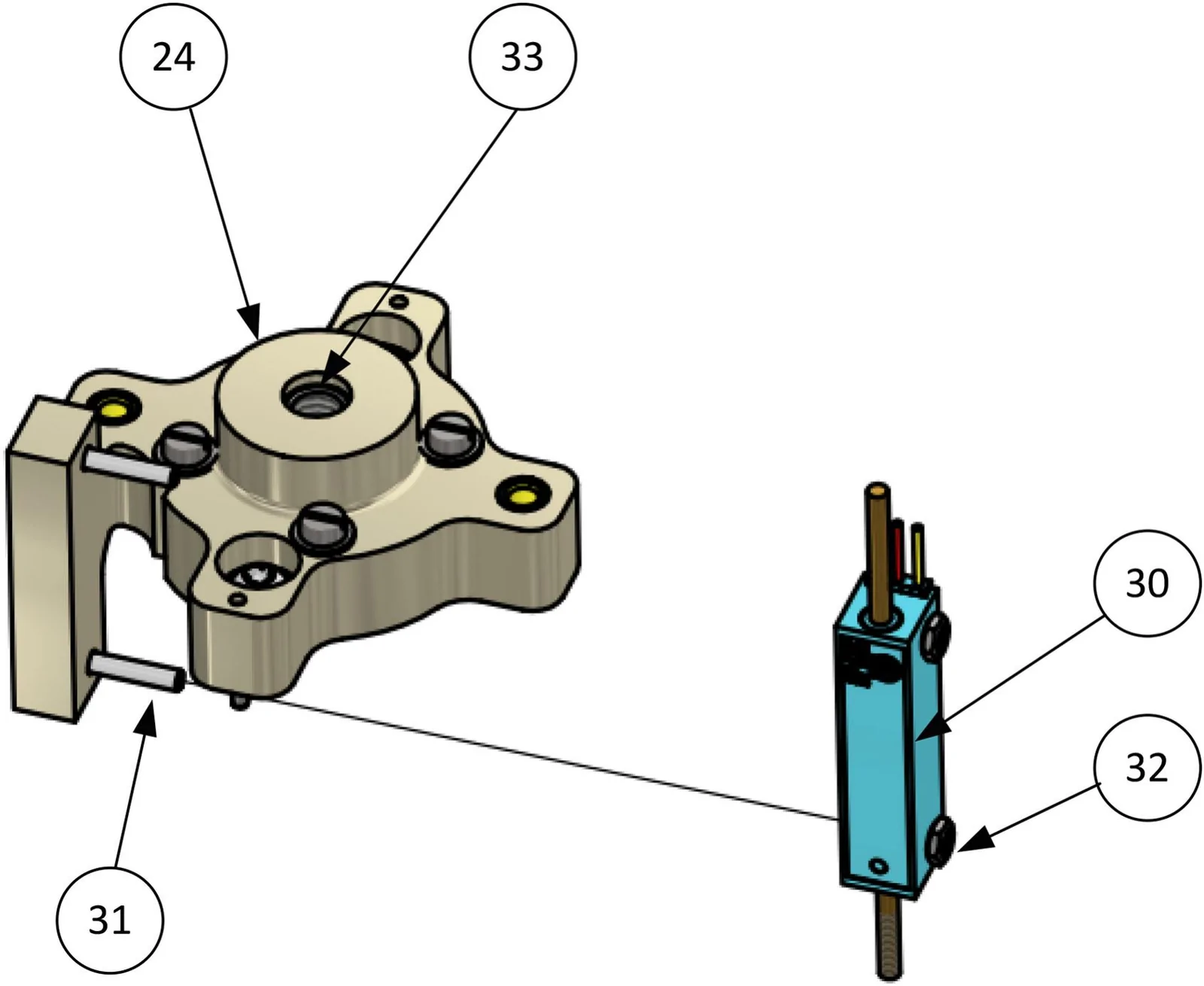
Assembly of the pneumatic cylinder potentiometer onto the Base9.

The potentiometer (“30”) is attached to the Base9 (“24”) using two screws (“31”) and two nuts (“32”). The vertical potentiometer is useful for measuring the position of the MobileFrame15 with respect to the CentralPart12 (i.e., the piston rod position of the pneumatic cylinder). This position feedback enables the future implementation of closed-loop motion control for the pneumatic cylinder within the gripper control firmware.

Then, the nut (“33”) is mounted in its final position, and it will connect the Base9 to the automatic tool changer in Step 13.

**Step 11 – Base9 and Flange2 assembly – (** Fig. 18 **)**

**Fig. 18:**
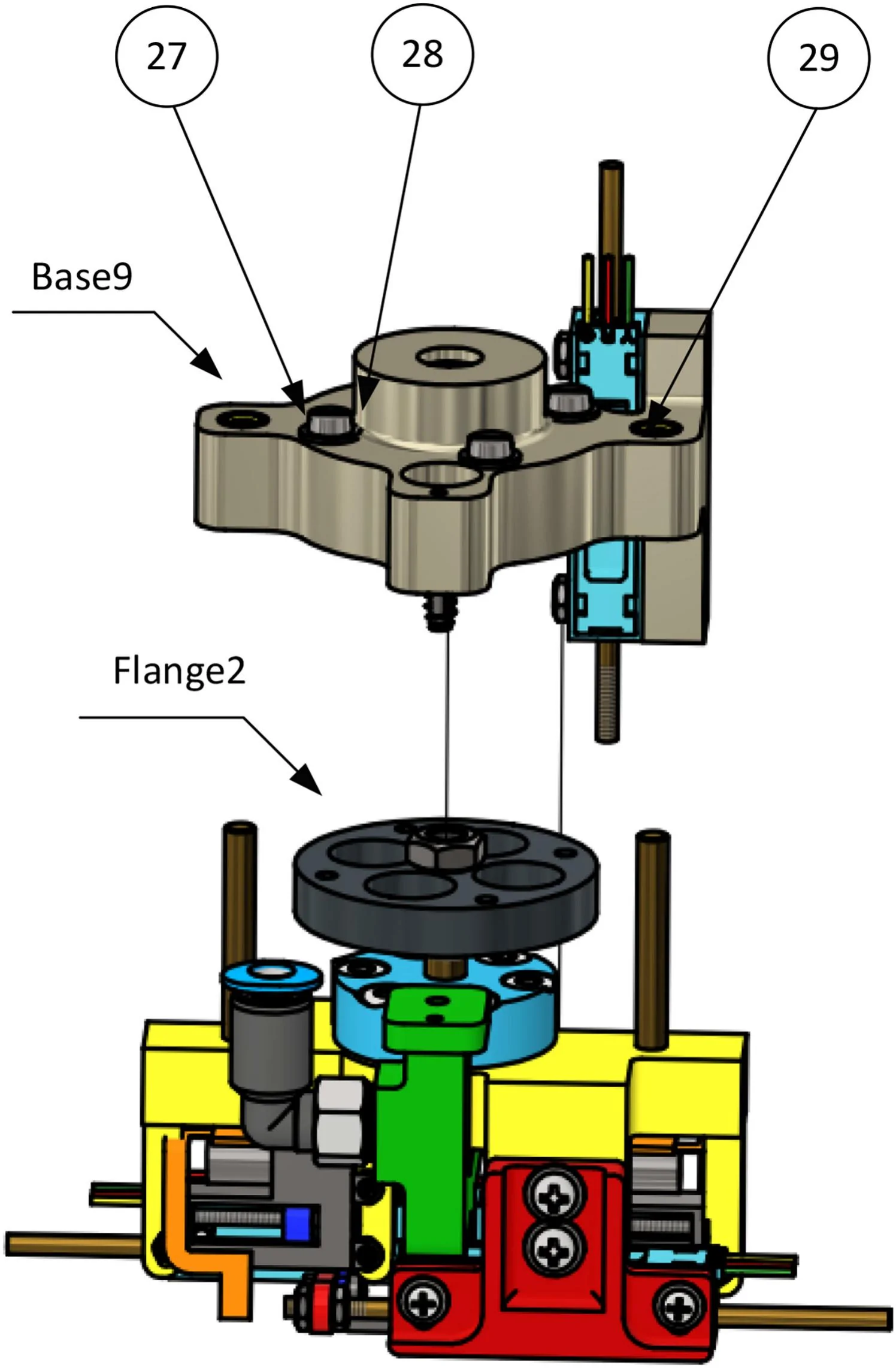
Screwing Base9 to Flange2.

Four screws (“27”) and four washers (“28”) connect Base9 sub-assembly to Flange2 (“34”). Two bearing bushes (“29”) are inserted inside the corresponding holes in the Base9 sub-assembly. These bearing bushes guide the vertical brass pins (“2”) of the MobileFrame15 assembly (“1”), enabling a low-friction vertical motion actuated by the pneumatic cylinder.

**Step 12 – Base9 and CentralPart12 assembly – (** Fig. 19 **)**

**Fig. 19:**
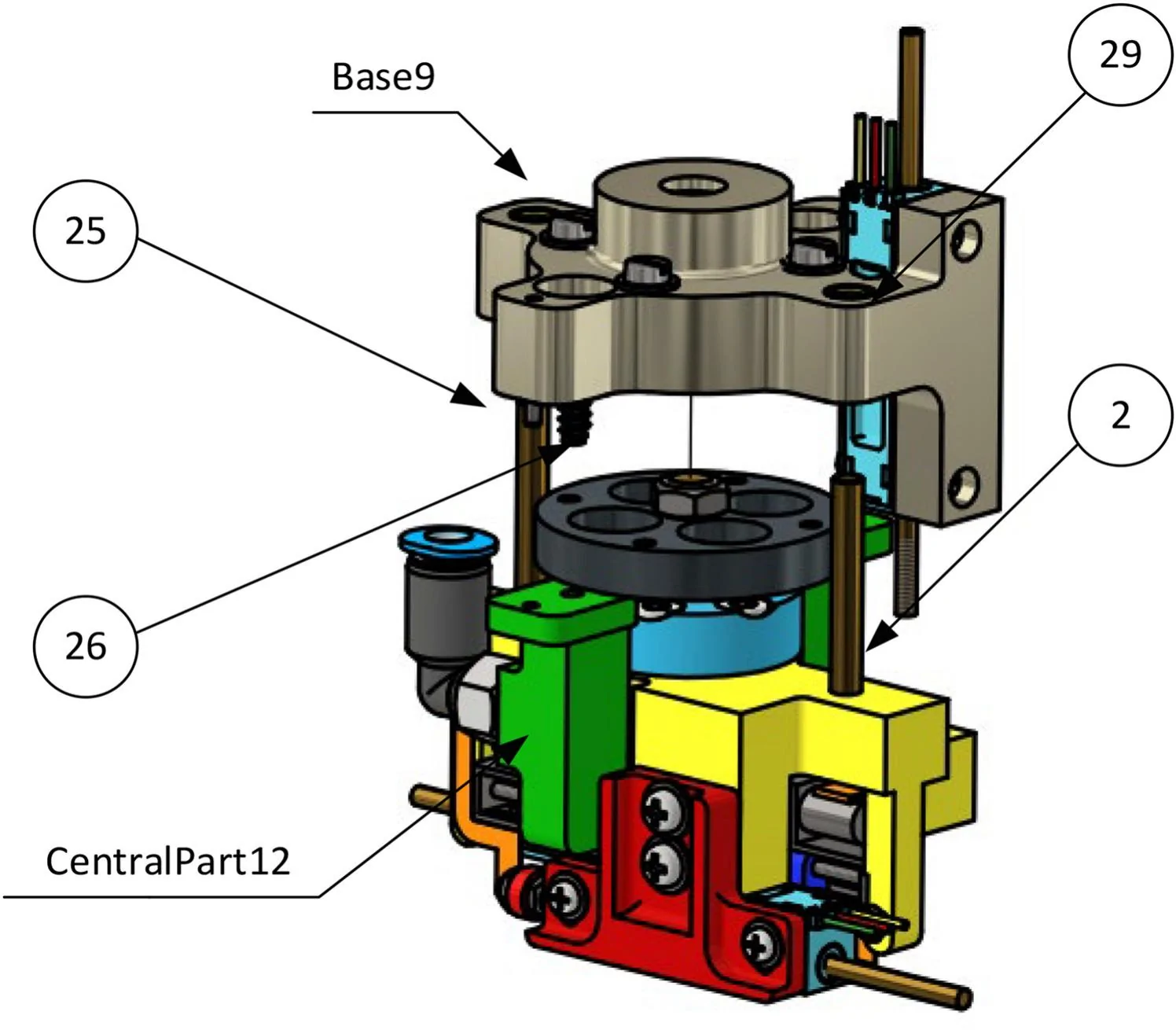
Joining the Base9 and CentralPart12.

In this step, the Base9 sub-assembly (that is already screwed to Flange2 in Step 11) is attached to the CentralPart12 sub-assembly. It is essential to pay attention to the alignment of the parts during this operation. Two pins (“25”) are used to align Base9 with CentralPart12 and two polymer screws (“26”) attach these sub-assemblies. This ensures that the two vertical brass pins (“2”) of the MobileFrame15 are properly aligned with the two corresponding bearing bushes (“29”). Moreover, the guided push rod of the vertical potentiometer is fastened with a nut to the MobileFrame15 (i.e., the yellow part).

**Step 13 – Robot interface assembly– (** Fig. 20 **)**

**Fig. 20:**
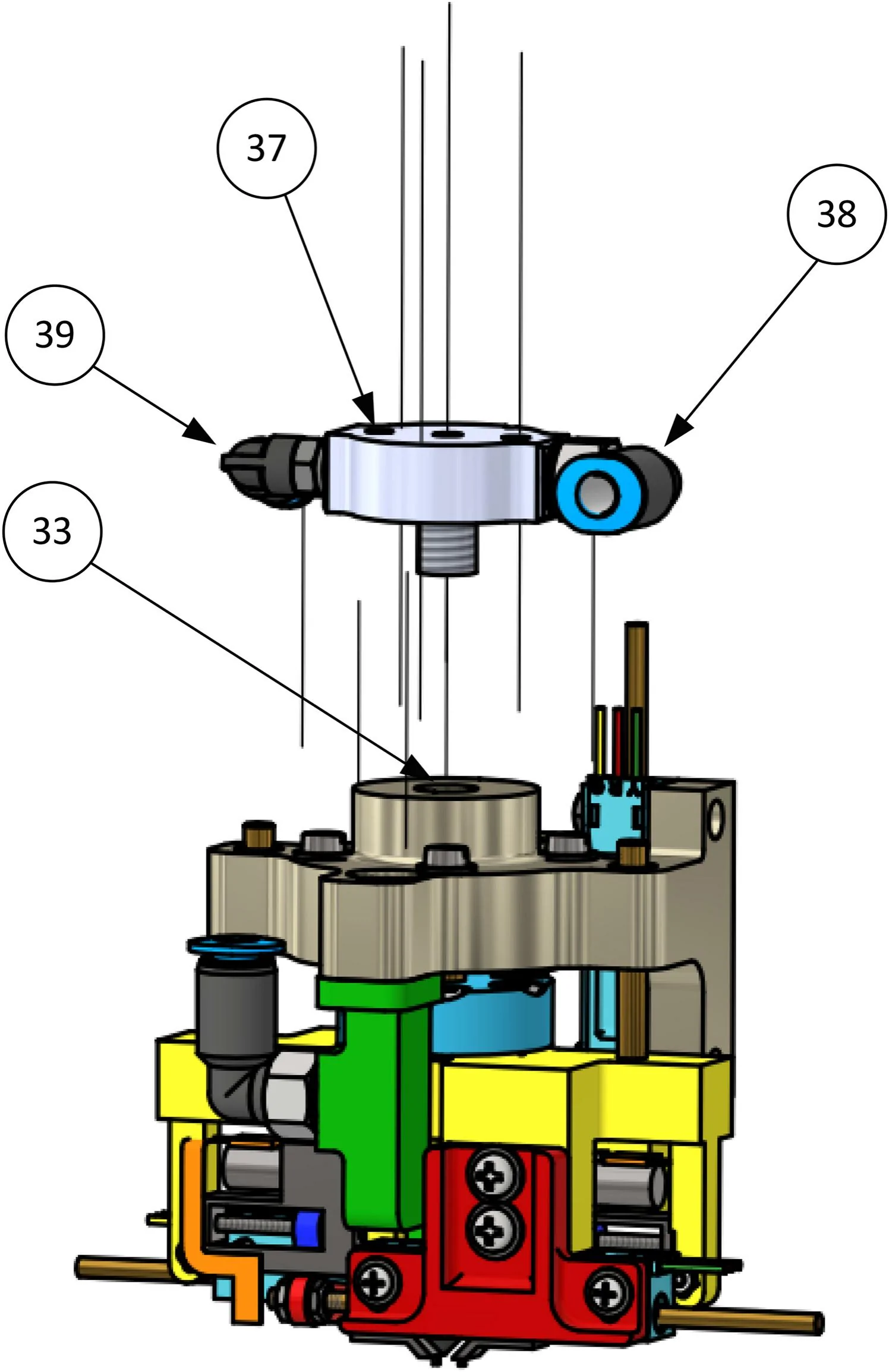
Assembly of the Robot Interface.

In this step, the Robot Interface (“37”) is attached to the gripper by screwing its male M6 thread into the nut (“33”) of the Base9.

Two push-in L pneumatic fittings are integrated into the Robot Interface:

• the former (“38”) is used for the vacuum supply of the suction cup;
• the latter (“39”) is used for supplying compressed air to the pneumatic cylinder.

**Step 14 – Assembly of the tool changer's bottom part – (** Fig. 21 **)**

**Fig. 21:**
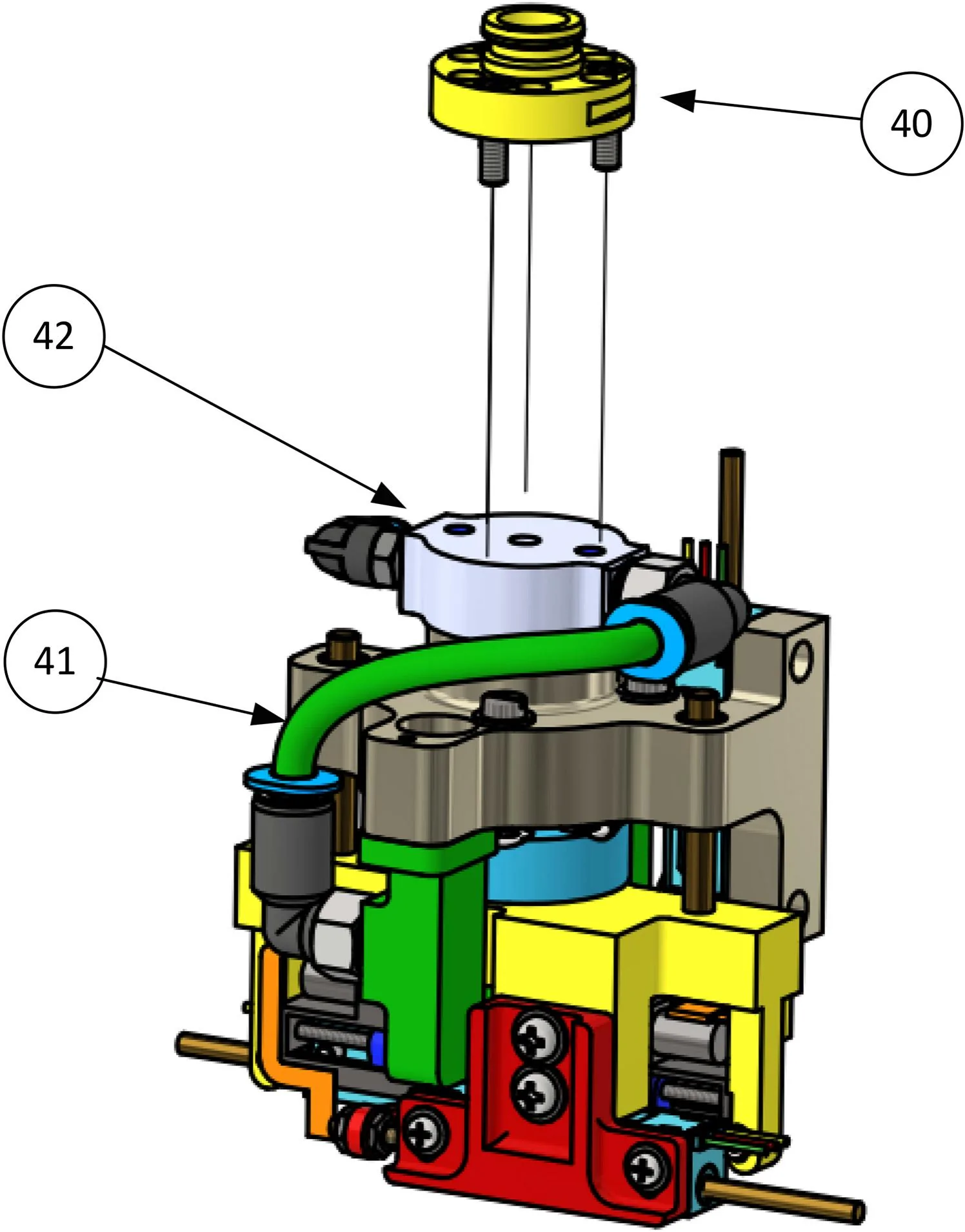
Assembly of the tool changer's bottom part and pneumatic and electrical connections.

At this step, the bottom part (“40”) of the tool changer can be assembled, and the two pneumatic connections can be installed:

• the former (“41”) is used for the vacuum supply;
• the latter (“42”, hidden by the Robot Interface in Fig. 21) is used for supplying compressed air to the pneumatic cylinder.

Moreover, the electrical connections of the miniature linear stages and potentiometers can be connected to the control system in this step.

**Step 15 – Mounting the micro-gripper on the tool changer’s top part – (** Fig. 22 **)**

**Fig. 22:**
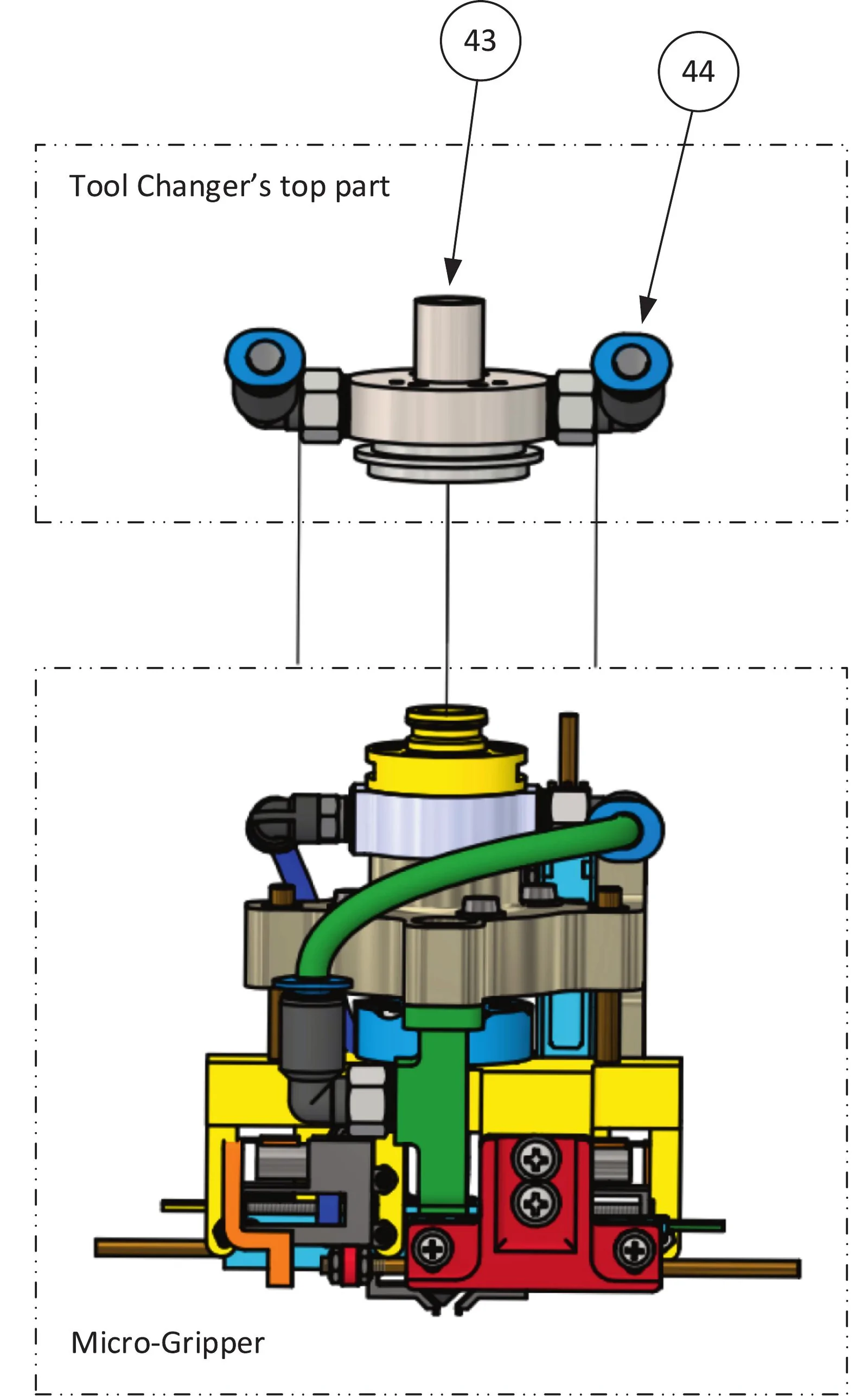
Final Assembly: the micro-gripper is mounted on the tool changer’s top part.

In the final step, the micro-gripper is mounted on a robot. The top part (”43”) of the tool changer, fixed to the robot flange, is coupled with the bottom part, fixed to the micro-gripper, by actuating the locking ring via the lever. The micro-gripper is fully assembled and integrated into the robotic manipulation system.

**Exploded view of the manufactured multi-mode hybrid micro-gripper – (** Fig. 23 **)**

**Fig. 23:**
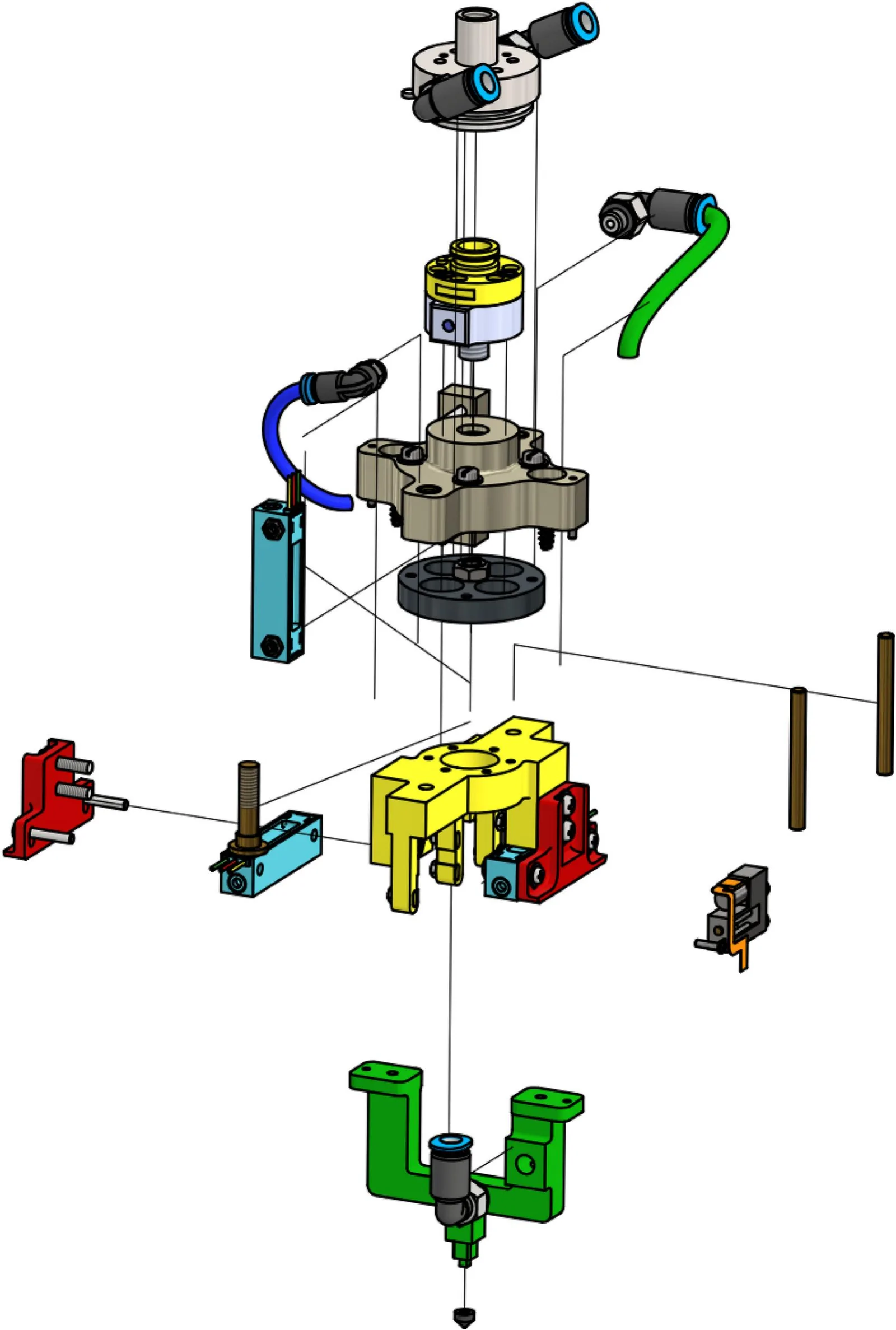
Exploded View of the manufactured micro-gripper.

## 6 Operational instructions

The micro-gripper is designed to operate as a robot end-effector to perform pick-and-place tasks of SMT components for PCB assembly applications. Any other use is not compliant with its design scope. Under these specified conditions, the designed tool presents no potential safety hazards.

To operate the micro-gripper, a set of preliminary connections must be configured. They are illustrated in Fig. 24 and described in the following:

1. mechanical attachment (i.e. black arrow in Fig. 24): the micro-gripper must be installed on a robot flange, preferably using an automatic tool changer (as shown in steps 14 and 15 of Section 5);
2. pressure connection (i.e. blue arrow): the micro-gripper must be connected to the regulated air pressure source, according to the pneumatic schematic (refer to Section 3);
3. vacuum connection (i.e. green arrow): the micro-gripper must be connected to the vacuum ejector, according to the pneumatic diagram (refer to Section 3);
4. linear stage electrical connection (i.e. red arrow): the stepper motors of the stages must be connected to the stepper motor drivers according to the electronic schematics of the gripper control system (refer to Section 3);
5. potentiometer electrical connection (i.e., purple arrow): the linear potentiometers must be connected to the analogue input of the gripper control system according to the electronic schematic (refer to Section 3).

**Fig. 24:**
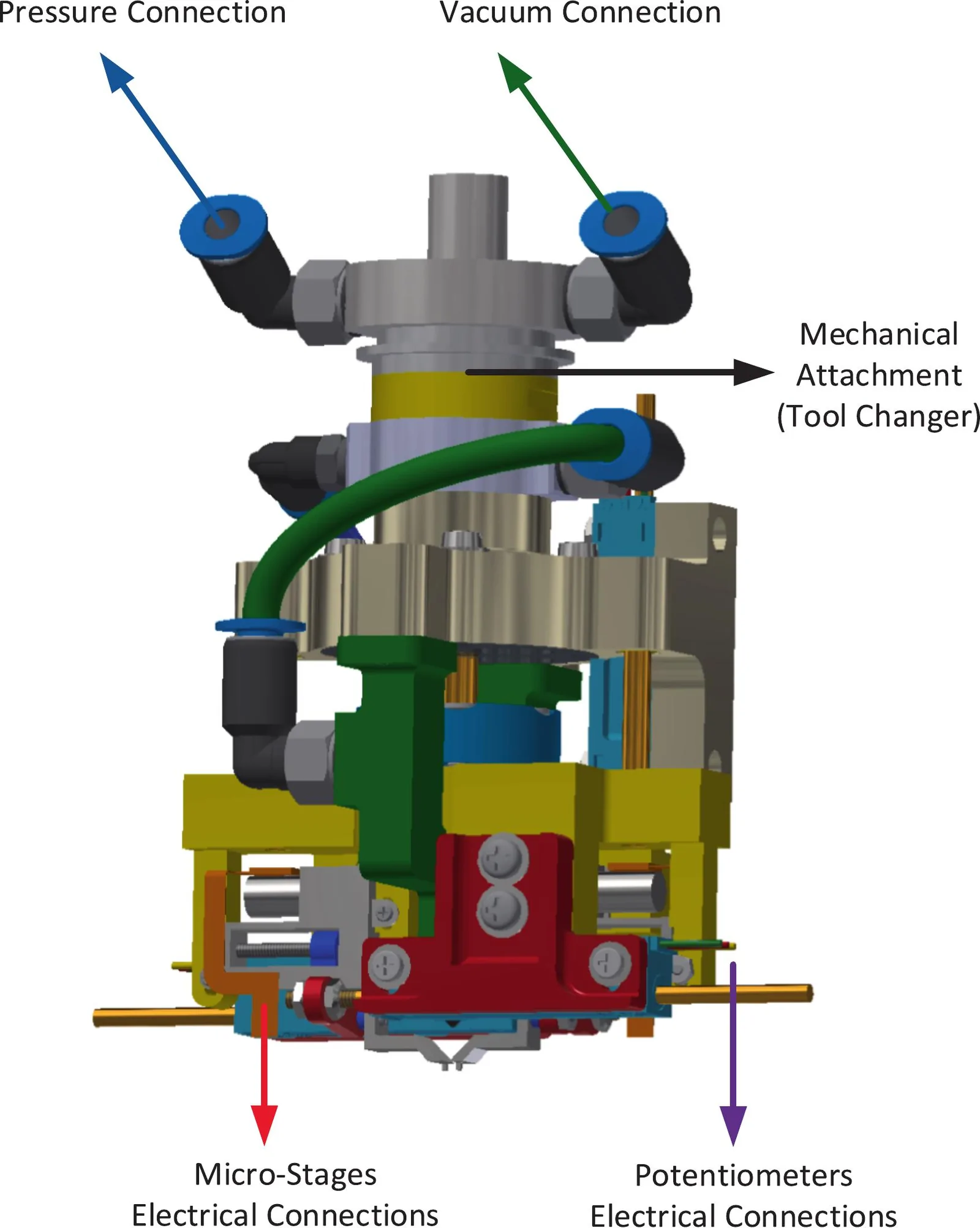
Overview of the connections needed to operate the micro-gripper.

Once all the above connections are completed, the micro-gripper is ready for grasping operations.

Three operational modes are supported:

• friction grasping: the component is gripped exclusively by the two micro-fingers;
• vacuum grasping: the component is gripped exclusively by the suction cup;
• hybrid grasping: both the micro-fingers and the suction cup are used simultaneously.

The following paragraphs illustrate these modalities.

### 6.1 Friction Grasping

The steps to follow to pick an SMT component exploiting the friction grasping, as shown in Fig. 25, are:

1. the friction grasping tool (i.e., MobileFrame15) is moved down to the bottom position by the springs of the pneumatic cylinder (or by the weight force of the mobile part). It is maintained in this position until the grasping operation is completed;
2. the robot approaches the electronic component to be picked. This approach takes place along the vertical direction, ensuring that the micro-gripper is precisely positioned in correspondence with the planar position (i.e. X and Y) of the component barycenter;
3. the robot moves down the micro-gripper along the Z axis to reach the component grasping height;
4. the micro-gripper closes the fingers by actuating the two micro-stepper motors, allowing it to grasp the component;

**Fig. 25:**
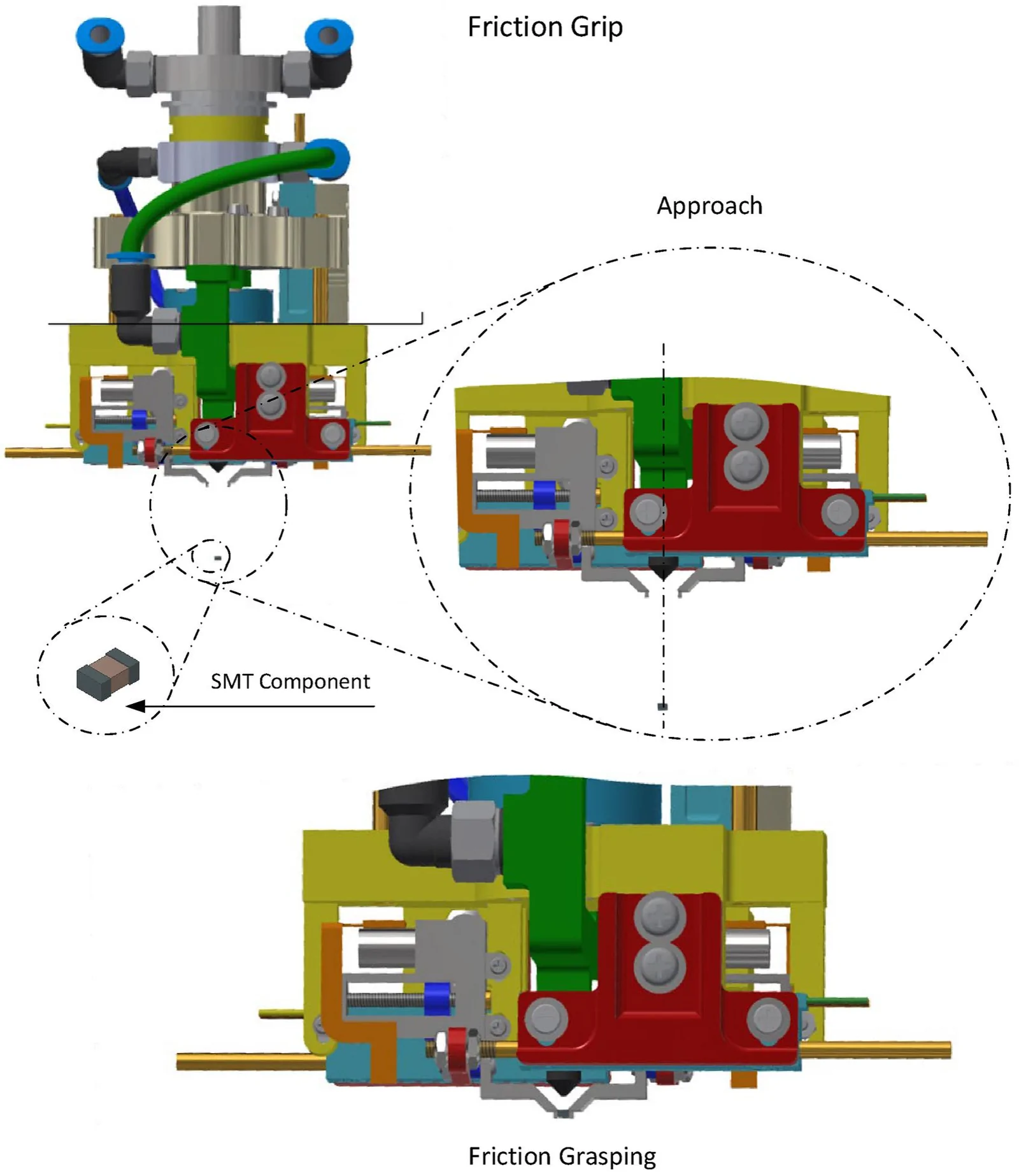
Friction grasping: general view and details of the fingers and the SMT component.

To release the SMT components, the operations to perform are:

5. the robot moves the gripper to the precise planar position (i.e. X, Y) where the SMT component must be released (i.e. the barycenter of the corresponding pads on the PCB);
6. the robot moves down the micro-gripper along the Z axis to reach the component releasing height;
7. the micro-gripper opens the fingers, and the SMT component is released;
8. the robot moves up the micro-gripper, and the following pick-and-place cycle can start.

### 6.2 Vacuum Grasping

The steps to follow to pick an SMT component exploiting the vacuum grasping, as shown in Fig. 26, are:

1. the friction grasping tool (i.e., MobileFrame) is moved up to the top position by a pneumatic cylinder. It is maintained in this position until the grasping operation is completed;
2. the robot approaches the electronic component to be picked. This approach takes place along the vertical direction, ensuring that the micro-gripper is precisely positioned in correspondence with the planar position (i.e. X and Y) of the component barycenter;
3. the robot moves down the micro-gripper along the Z axis to reach the component grasping height;
4. the micro-gripper supplies the vacuum to the suction cup, allowing it to grasp the component.

**Fig. 26:**
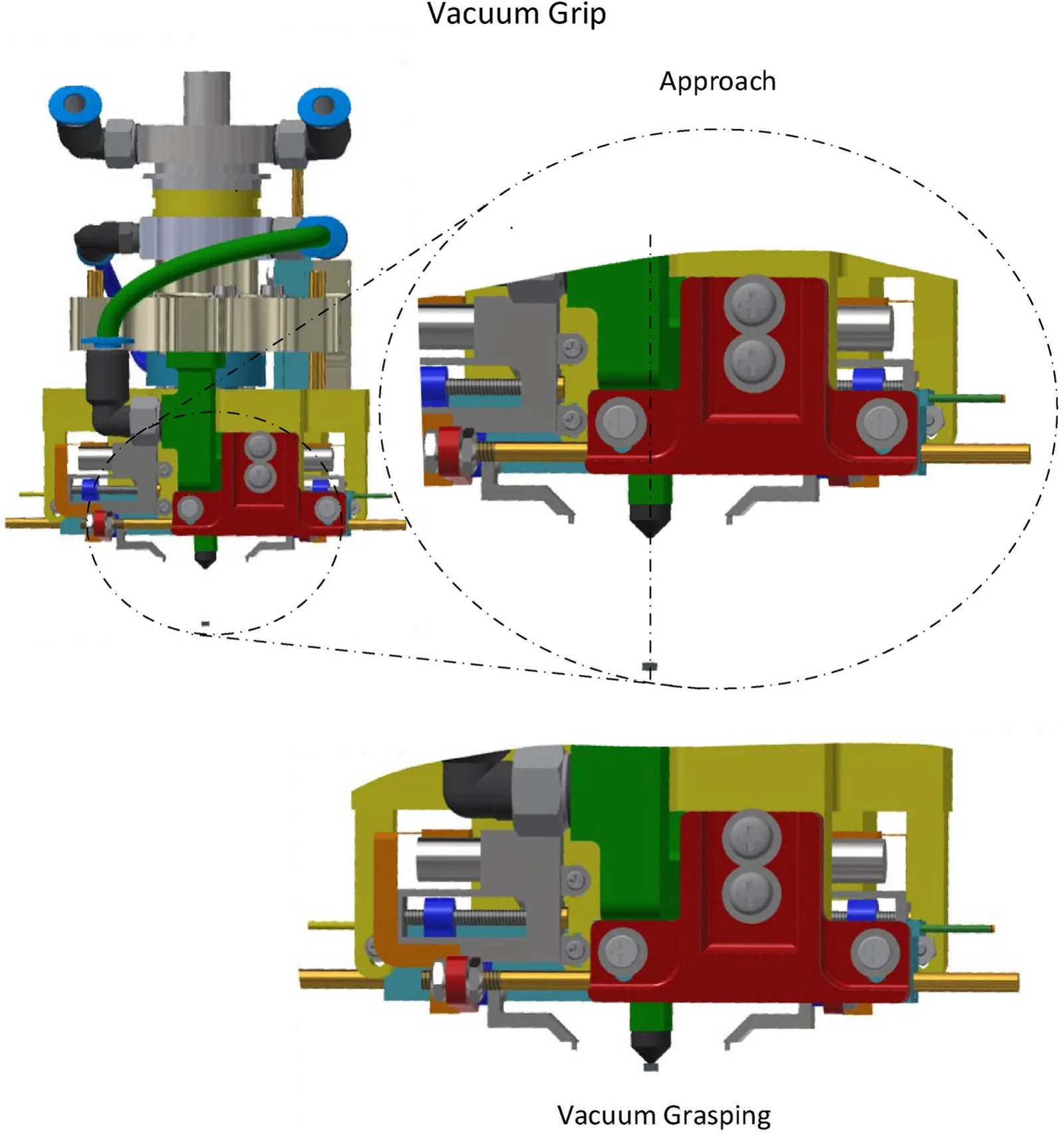
Vacuum grasping: general view and details of the micro suction cup and the SMT component

To release the SMT component, the operations to perform are:

5. the robot moves the gripper to the precise planar position (i.e. X, Y) where the SMT component must be released (i.e. the barycenter of the corresponding pads on the PCB);
6. the robot moves down the micro-gripper along the Z axis to reach the component releasing height;
7. the vacuum is turned off (i.e. atmospheric pressure is restored inside the suction cup), and the SMT component is released;
8. the robot moves up the micro-gripper, and the following pick-and-place cycle can start.

### 6.3 Friction and Vacuum Grasping (Hybrid Mode)

The hybrid operational mode combines the two previously described gripping methods, as shown in Fig. 27. The first steps correspond to those performed to pick a SMT component with the friction grasping (i.e. steps 1, 2 and 3). Once the friction grasping is performed, the friction grasping tool (i.e. MobileFrame) is lifted up to place the SMT component in contact with the suction cup. When the vacuum grasping is activated, the hybrid approach is performed, since the component is gripped simultaneously by both grasping tools.

**Fig. 27:**
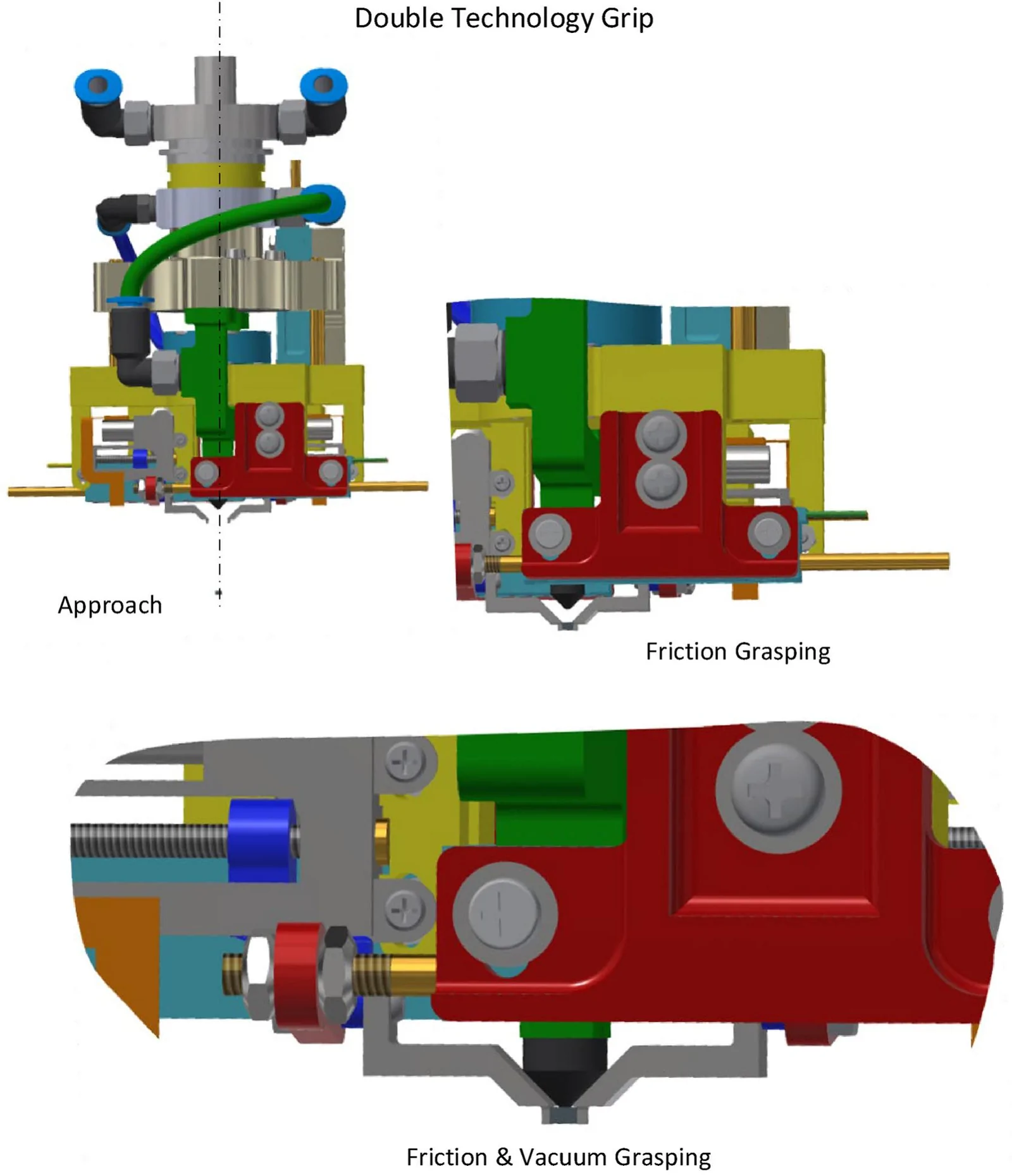
Step of hybrid grasping: approach and friction & vacuum grasping.

To release the component (once the micro-gripper is moved to the release position by the robot and the component has been soldered on the corresponding PCB pads), the described grasping procedure is executed in reverse order: the vacuum is turned off, and the two fingers are opened to release the component. Additionally, to release micro-scale components, it is possible to synchronise the upward motion of the fingers with their opening movement, while simultaneously deactivating the vacuum. In this way, the suction cup acts as a pushing plug on the component that facilitates its release, overcoming the micro-scale adhesion forces. Therefore, the hybrid approach incorporates an active releasing mechanism that exploits the fingers' vertical motion relative to the suction cup during the releasing phase.

Finally, the robot moves up the micro-gripper, and the following pick-and-place cycle can start.

## 7 Validation and Characterisation

### 7.1 Control System Calibration

#### 7.1.1 Calibration Procedure for Position Sensors

Position feedback for the closed-loop control of the linear stages in friction gripping is obtained by accurate linear potentiometers with a measurement range of 11 mm. The 12-bit ADC integrated within the microcontroller achieved a position resolution of 2.68 µm. To enhance measurement accuracy and reduce measurement noise of the linear potentiometers, a second-order IIR low-pass filter with a cutoff frequency of 50 Hz was designed, using the MATLAB Filter Designer Toolbox. Adopting the designed filter, the standard deviation of the sensor signals was minimised by 87%, reducing it to 2 µm in the worst case.

The Mitutoyo Digimatic Indicator ID-C112B was used to measure the stroke of the linear stages. This measurement resulted in a maximum movement range of 9119 µm for the first finger and 9086 µm for the second finger.

The sensors' linear calibration is performed via an automated task implemented in the designed control firmware. At the initialisation phase or when receiving a calibration command, the controller performs the calibration process, acquiring the potentiometer raw values from the ADC at the motion range extremes (i.e., limit switches). Knowing the total stroke of the linear stage, the sensor sensitivity is calculated to convert the ADC raw measurements into position values.

#### 7.1.2 System modelling and PID tuning

The controller of the friction grasping uses a closed-loop motion control method to reach the target position of the linear stages (i.e. fingers) within a defined steady-state error limit, effectively addressing common issues in stepper motor control, including step loss, mechanical backlash, and energy efficiency. The system adopts a decentralised approach, incorporating a position controller for each linear stage. Each finger control loop includes a PID controller that generates step pulse commands for the stepper motor of the linear stage based on position error. A back-calculation anti-windup mechanism was implemented to compensate for the integrator term of the PID controller. A speed feedforward was also integrated into the PID output to enhance the position setpoint tracking.

The PID controller tuning method started with linear system identification, involving different open-loop motion tests for each axis (i.e. a time-domain step-response identification). For each test, a trapezoidal speed profile was set as input to the linear stages, and the reached output positions were obtained using the calibrated linear potentiometers. The tests were carried out at motion speeds ranging from 3 mm/s to 24 mm/s, with durations varying from 0.4 to 3 seconds. An acquisition sample time of 0.5 ms was used to acquire the positions of the linear potentiometers. An example of a motion cycle for the system identification is shown in Fig. 28.

**Fig. 28:**
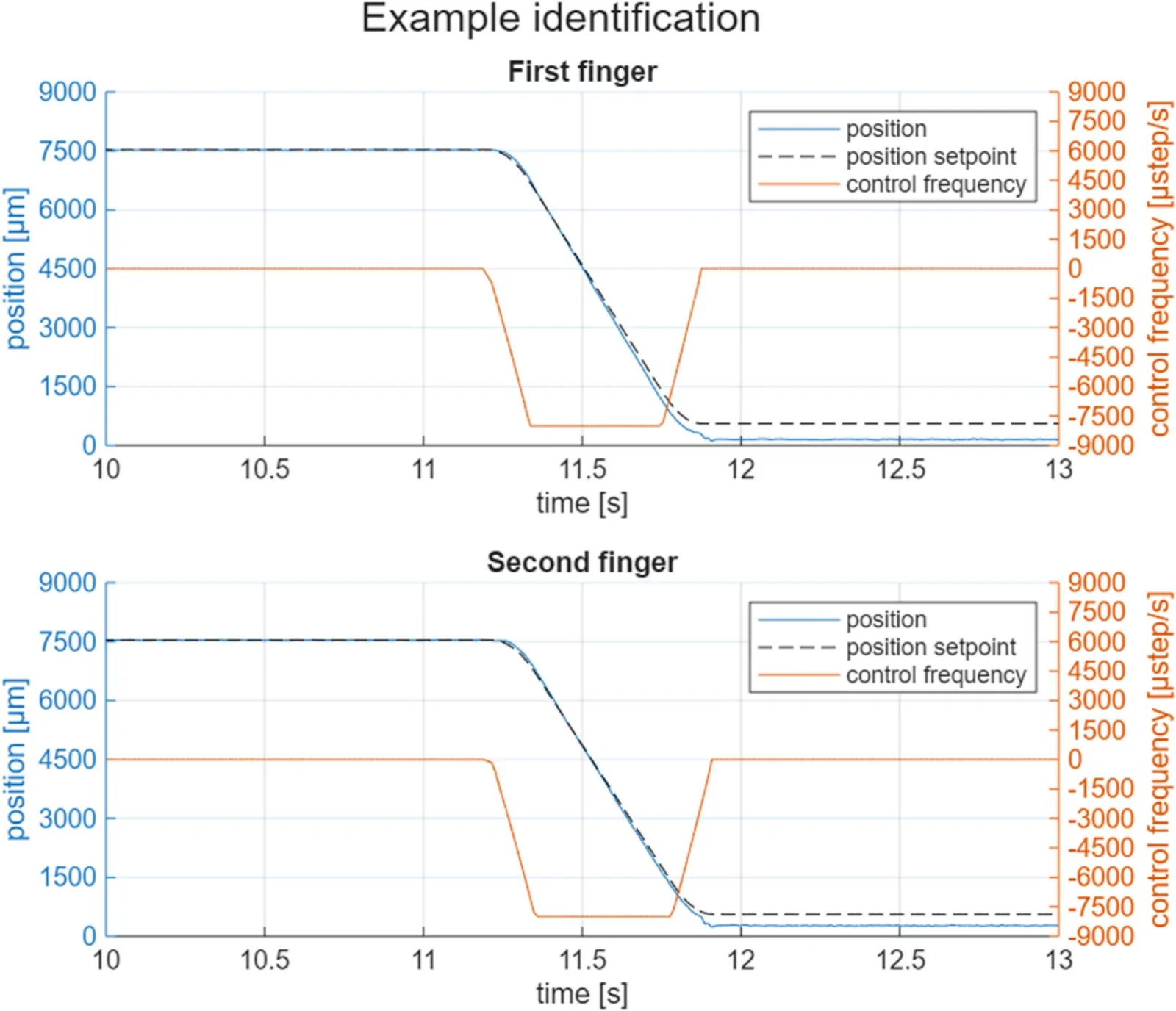
Plots of an identification cycle with a maximum step frequency of 8000 µstep/s.

Using MATLAB's System Identification Toolbox, first-order transfer functions were determined for the linear stages (i.e. fingers). The equations and parameters are presented in Eq. (7) and Table 3. The models were validated by additional motion cycles, achieving a minimum accuracy of 96% in fitting the systems’ dynamics.

(7) $$P\left(s\right)=K\cdot \frac{1}{1+T\cdot s}$$

**Table 3:** First-order model parameters for both fingers.

**Finger**	**T**	**K**
1	24.15	853.1
2	73.49	2529.4

The MATLAB PID Tuner Toolbox was employed for tuning the PID controller (see Eq. (8) for each axis. The PID tuning focused on maximising robustness to improve setpoint tracking performance (e.g., minimising overshoots) and optimising noise rejection. The parameters, shown in Table 4, were carefully tuned for each axis to achieve a balance between responsiveness and stability, starting from the models developed in the system identification.

(8) $$R_{\mathrm{PID}}\left(s\right)=K_{P}+\frac{K_{i}}{s}+K_{d}\cdot s$$

**Table 4:** PID controller parameters for both fingers.

**Finger**	$K_{P}$	$K_{i}$	$K_{d}$
1	0.1020	0.0064	0
2	0.1223	0.0090	0

The control system calculates the motion control variable only when errors exceed a threshold, i.e. the positioning control tolerance. The calibration of this positioning tolerance involved setting an acceptable range for positioning inaccuracy to prevent chattering in the control variable that induces unnecessary displacements, which could damage the motor and reduce its lifetime. The control tolerance was determined by evaluating the maximum standard deviation of position feedback measurements, the step size of the stepper motor, and the mechanical backlash of the linear stages. The control tolerance was established at 50 µm, carrying out experimental closed-loop motion tests.

### 7.2 Demonstration of Hardware Operation

#### 7.2.1 Grasp and Release Operations

The grasp and release experimental tests demonstrate the gripper's capability and adaptability to manipulate different SMT components, exploiting its three operational modes: friction-based, vacuum-based, and hybrid grasping. A Ball Grid Array (BGA) component and a micro-resistor were used to perform these manipulation tests. The chosen samples represent the maximum and minimum sizes of the components that the designed gripper can manipulate. A total of 30 test cycles of grasp and release operations for each component and each grasping technology were executed.

For friction grasping, the control system exploited the designed closed-loop motion algorithm to ensure precise positioning and synchronisation of the fingers, while for vacuum grasping, the two-state relay controls were used to drive the 3-2 solenoid valve and the proportional pressure regulator of the vacuum ejector connected to the suction cup. The hybrid grasping, combining the two grasping techniques, also employed the pneumatic cylinder to control the vertical movement of the fingers. The two-state relay controls were used to drive the 3-2 solenoid valve and the proportional pressure regulator of the pneumatic cylinder.

The gripper was able to manipulate (i.e. grasp, hold, and release) with high accuracy the BGA components with dimensions of 8 × 6 × 0.8 mm, using all its grasping techniques as can be seen in Fig. 29a (i.e. friction-based), 29c (i.e. vacuum-based) and 29e (i.e hybrid approach). Each technique achieved a reliability of 100% in picking and releasing the BGA.

**Fig. 29:**
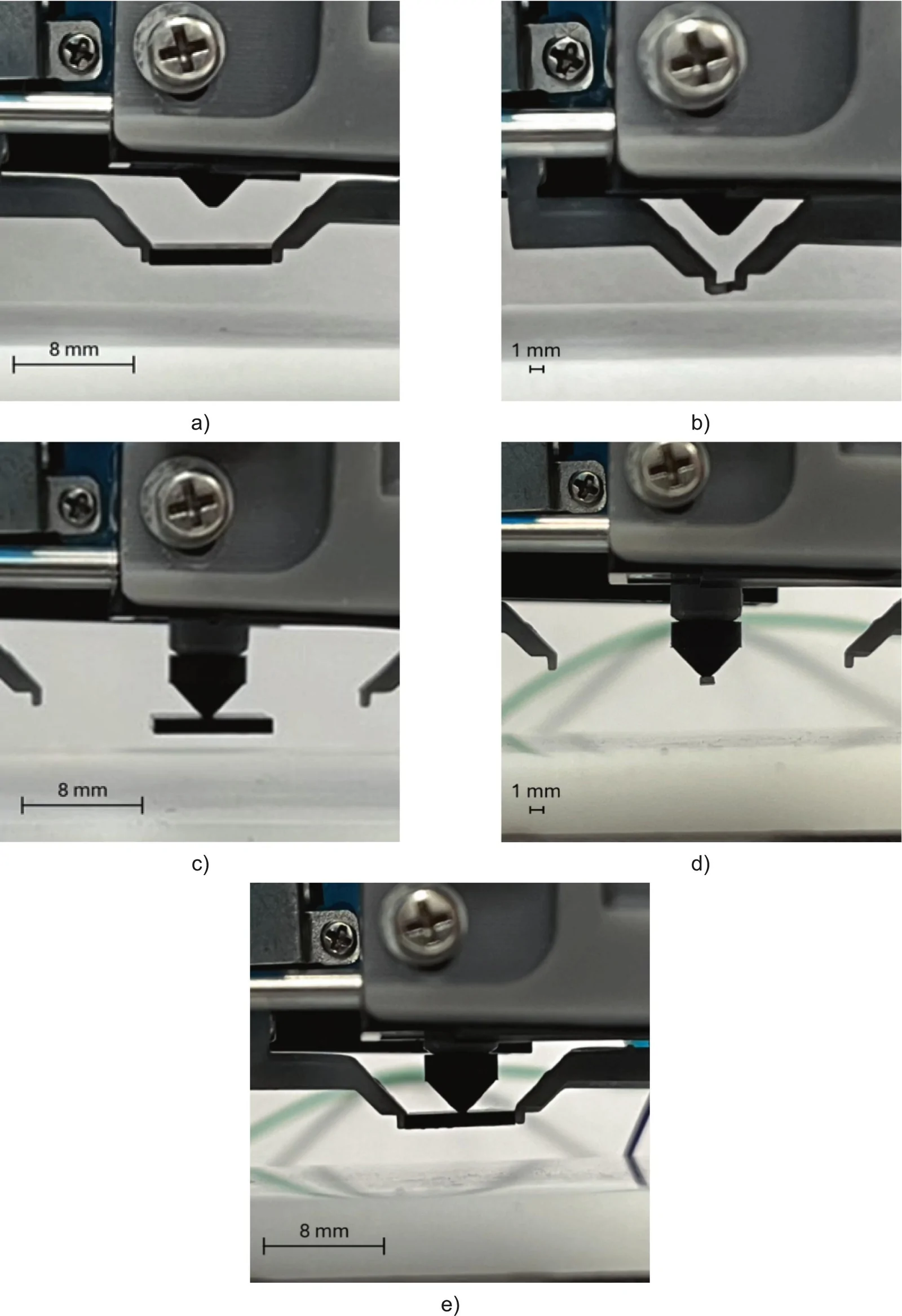
Grasping phase of the grasp and release tests for the BGA and 1005 resistor using the different grasping modes: (a) BGA – friction; (b) 1005 resistor – friction; (c) BGA – vacuum; (d) 1005 resistor – vacuum; (e) BGA – hybrid approach

The 1005 resistor with dimensions of 1 × 0.5 mm was effectively grasped, held, and released with high precision, exploiting the friction (as shown in Fig. 29b) and the vacuum-based (as shown in Fig. 29d) techniques, achieving a picking and releasing reliability of 100% for both methods. The hybrid approach failed to manipulate this component due to mechanical interference between the fingers and the suction cup. This limitation could be overcome by modifying their shape designs.

The tests performed with the designed gripper have shown its capacity to manipulate and assemble efficiently, accurately and reliably various types of SMT components with dimensions in the specific design range. For these reasons, the gripper is suitable to be mounted on a robotic system to perform automated micro/meso-manipulation tasks for PCB assembly with SMT components.

The friction grasping technique is ideal for components that need firm and accurate manipulation. However, it is less suitable for fragile components and requires more space for handling due to finger movements. This can be an issue for PCBs with high component density, causing interference and impact. The vacuum grasping is more suitable for applications where components require delicate and accurate handling, but it offers less holding stability than the friction technique because it applies only a monolateral force. The hybrid approach provides the most holding stability and release precision, since the vertical movement of the fingers with respect to the suction cup can act as an integrated releasing mechanism, but it is not yet suitable for components with dimensions below the millimeter with the current designs of its grasping tools (i.e. the fingers and suction cup).

Therefore, the designed multi-mode hybrid micro-gripper contributes to the ongoing efforts to enhance the reconfigurability and flexibility of robotic workstations in PCB smart manufacturing. It can effectively manage and manipulate the high density of components with different sizes and shapes that need to be assembled on the PCB, while also minimising set-up time.

#### 7.2.2 Friction grasping test

A series of experimental tests was carried out to analyse the positioning and synchronisation performance achieved by the closed-loop control system designed for the gripper fingers.

Firstly, the setpoint tracking performance of the designed closed-loop control of the two fingers was assessed.

As said above, a series of picking and releasing cycles of different sample SMT components (i.e., a BGA package with a size of 8 × 6 × 0.8 mm and a 1005 resistor with a size of 1 × 0.5 mm, both with a prismatic shape) was executed.

During each trial, setpoint tracking and finger motion synchronisation (i.e., the time delay error between finger motions) errors were measured. The corresponding error means and standard deviations were calculated.

A test cycle was designed to replicate a robotic pick-and-place task of SMT components for assembling on pads of a PCB substrate. Each trial involved accurate closed-loop positioning of both gripper fingers to grasp the component, followed by an upward movement of the micro-gripper executed by the robot, a downward movement, and concluded with a closed-loop motion of the grippers to release the component on the substrate with high accuracy. In an actual pick-and-place cycle, the downward motion of the gripper would usually be preceded by the positioning of the robot above the final release position. However, this step was omitted in these tests, as it is not relevant for evaluating the gripper’s functionality.

The tests exhibited the gripper's capability to manipulate components with dimensions compliant with the specific design range using friction grasping technology. Performance assessment shows a reliable setpoint tracking with a maximum positioning error of ±50 µm (i.e., equal to the positioning control tolerance) for both fingers and synchronised movements of the fingers with a maximum time delay error under 30 ms. The setpoint tracking plots for a grasp and release test cycle of the BGA component are shown in Fig. 30. This test consisted of relative displacements (i.e. opening and closing) of 5.2 mm for the first finger and 4.9 mm for the second finger, with a maximum step frequency of 5 kHz in both movement directions for both fingers.

**Fig. 30:**
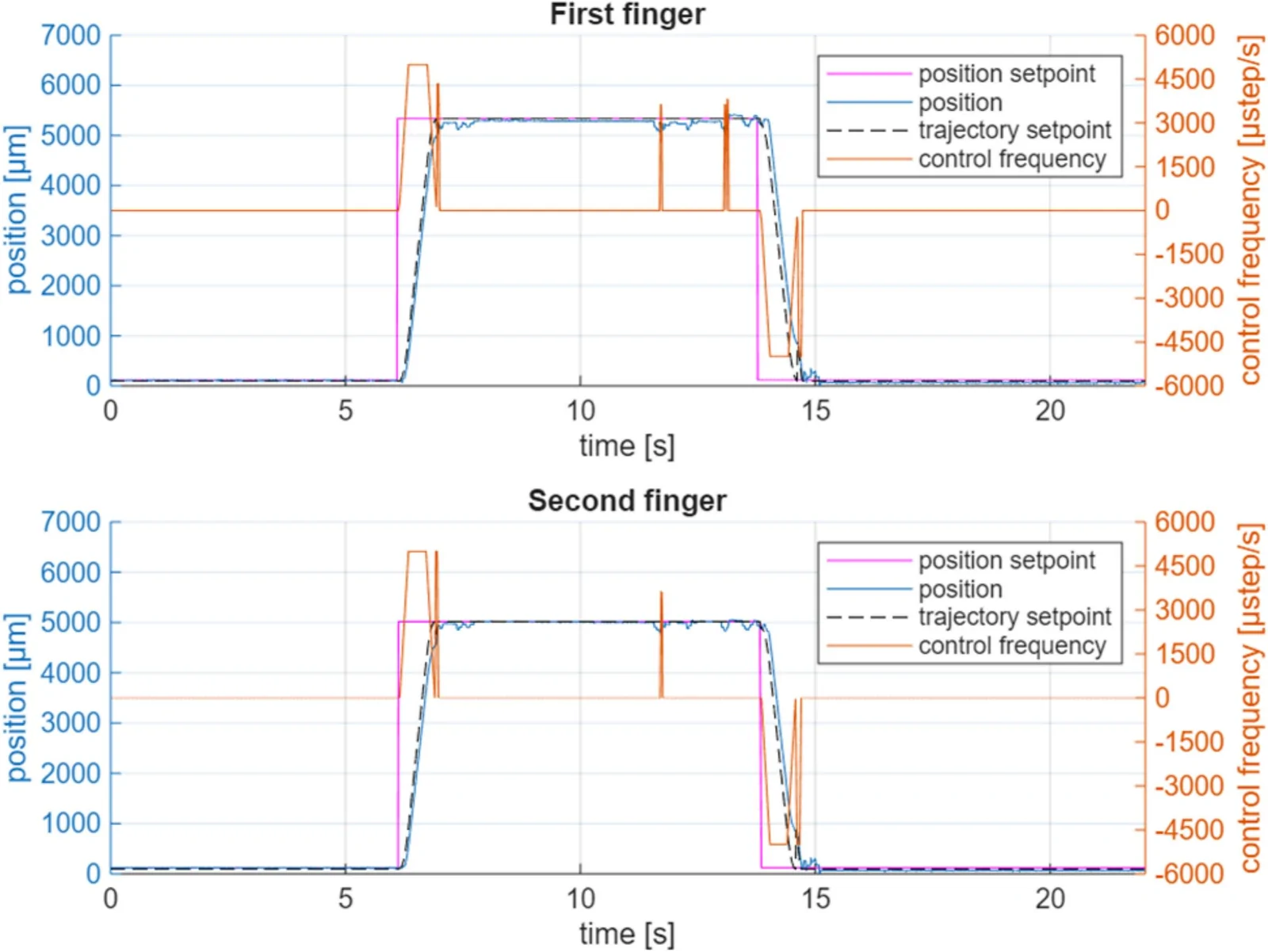
Plots of a grasping and releasing test of the BGA package exploiting the designed closed-loop motion control of the fingers.

#### 7.2.3 Object Detection (Stall)

The second experiment focused on assessing the performance of the stall detection (or object detection) functionality in the finger motion closed-loop control. The stall detection strategy was primarily introduced to prevent the motors from continuously applying force when further motion is mechanically impossible due to an obstacle, thereby avoiding potential damage. In this work, the stall threshold was set equal to the estimated mechanical backlash of the axis. As a result, the detection does not depend on the specific component being grasped, but rather on the commanded motion itself. Specifically, when the commanded displacement exceeds the stall threshold (i.e., the estimated backlash), a stall can be reliably identified. Conversely, if the commanded movement remains below this threshold, the linear stage concludes its displacement without stall detection intervening, even in the presence of an obstacle. To experimentally evaluate this functionality, multiple repetitions of picking and releasing test cycles were performed using an electrolytic SMT capacitor with a size of 6 × 8 mm and a cylindrical shape. The stall detection strategy was developed to detect either finger collisions with the environment during the releasing phase or to identify finger contact with the components or themselves in the grasping phase. It is possible to enable this control strategy independently or dependently for each finger. In the former case, when a stall is detected in one finger, only that finger will stop; in the latter case, both fingers will stop. The first option was chosen for both the picking and releasing phases in this test.

To grasp the component, a complete closing was commanded for both fingers (i.e. a relative movement of 5.6 mm, as the starting position of the fingers was partially opened). Once the fingers contacted the capacitor, they stopped independently due to the stall detection functionality and held the component in place due to the motor holding torque. During the releasing phase, a forced collision with a component mounted near the capacitor on the PCB was introduced by commanding a complete opening of the fingers (i.e. strokes of 9.1 mm). Once one finger contacted an obstacle, the control system detected the stall and stopped it, while the other finger completed its movement. The position tracking plots of a picking and releasing cycle of the capacitor with the stall detection functionality enabled are shown in Fig. 31. The maximum step frequency of the movement was set to 5 kHz for both fingers in both directions. It is worth to note, that when the stall occurred, the position and trajectory setpoints (i.e. respectively pink and dotted black lines in Fig. 31) continued increasing or decreasing until the position error exceeded the predefined stall threshold (i.e. a control system parameter). After reaching this threshold, the position setpoint was fixed at the current position, and the motion law was halted.

**Fig. 31:**
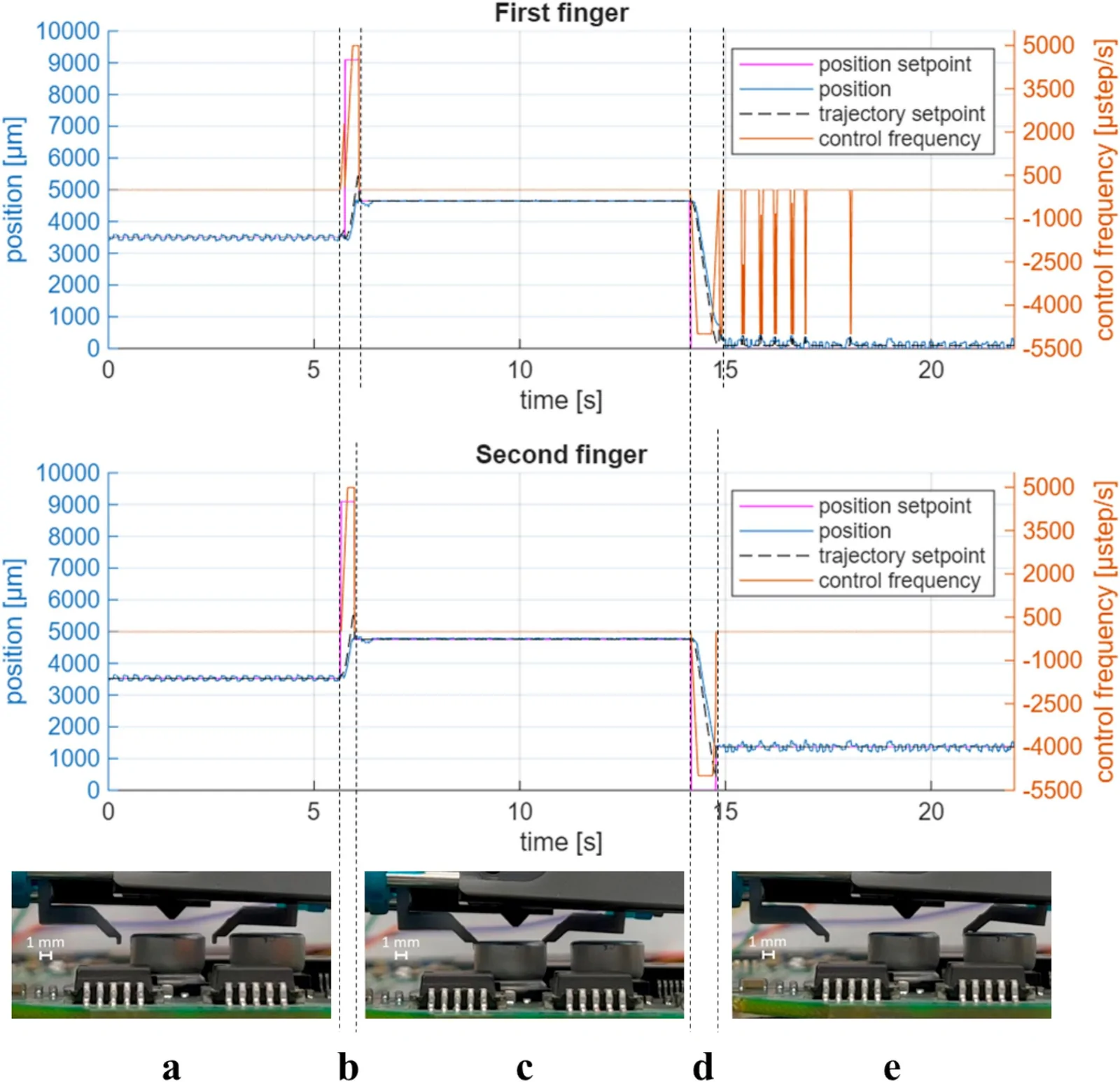
Plots of a grasping and releasing test cycle of the capacitor with the stall detection functionality enabled in the closed-loop motion control of the fingers. The motion can be divided into five phases: a) the linear stage motor is active while the controller remains idle, monitoring the position error and waiting for a command. b) upon receiving a command, the motion law is initiated. The position feedback (i.e. blue) follows the trajectory setpoint (i.e. dotted black) until a stall occurs. At this point, the position remains constant while the trajectory setpoint increases, since the commanded displacement is not achieved. Once the position error reaches the stall threshold, the motion is stopped and the setpoint is reset. This occurs for both fingers independently, and the stall is caused by contact with the grasped component. c) the motor is still active while the controller is idle, as in phase (a). d) a new command is executed. The first finger completes the commanded motion without obstacles, whereas the second finger collides with a nearby component and is stopped by the stall detection algorithm. e) the motor is active while the controller is idle. Minor position corrections are applied by the first finger's controller to compensate for noise or disturbances. (For interpretation of the references to colour in this figure legend, the reader is referred to the web version of this article.)

#### 7.2.4 Disturbance Rejection

A further test was performed to assess the disturbance rejection of the closed-loop motion control system of the fingers. Controlled external forces were applied to each gripper finger at two distinct time instants, and the system dynamic was analysed. When an external force was applied to a finger, the controller reacted by computing new motion laws. This adjustment continued until the position error stopped increasing, indicating that the external disturbance could have disappeared. The system then returned to its setpoint position following the last computed motion law. The new laws of motion were computed based on the configuration stored in the controller memory. When a motion law is calculated, a new motion starts from the actual position. The number of steps needed to restore the setpoint position is computed while avoiding an excessive starting speed. Fig. 32 shows the position tracking during the disturbance rejection test. The same dynamic behaviour was observed for both fingers, regardless of the motion direction (i.e., opening or closing). In Fig. 32, it is worth to note that when the external forces were removed, the last computed motion law was followed as expected for both fingers.

**Fig. 32:**
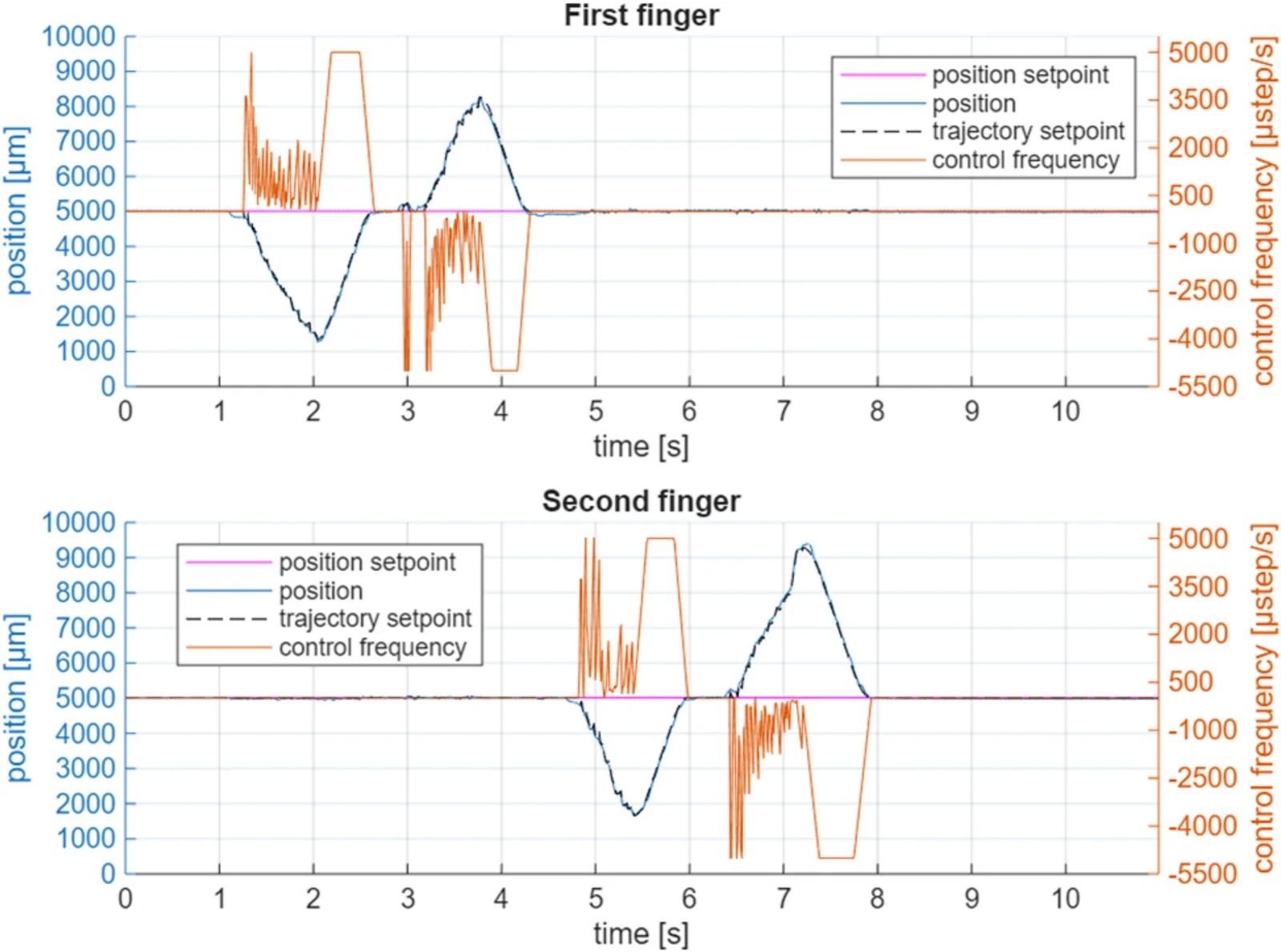
Plots of the disturbance rejection experimental test exploiting the designed closed-loop motion control of the fingers.

## CRediT authorship contribution statement

**Gianmauro Fontana:** Writing – review & editing, Writing – original draft, Validation, Software, Methodology, Investigation, Data curation, Conceptualization. **Nicola Iacono:** Writing – review & editing, Validation, Software, Data curation, Investigation. **Gabriele Papadia:** Writing – review & editing, Supervision, Project administration, Funding acquisition. **Simone Pio Negri:** Writing – original draft, Validation, Methodology, Conceptualization, Investigation.

## Funding

This work was partially funded by the spin-off of the University of Salento Advantech-LIKE S.r.l. (Lecce, IT).

## Declaration of competing interest

The authors declare that they have no known competing financial interests or personal relationships that could have appeared to influence the work reported in this paper.
